# Involvement of serum‐derived exosomes of elderly patients with bone loss in failure of bone remodeling via alteration of exosomal bone‐related proteins

**DOI:** 10.1111/acel.12758

**Published:** 2018-03-30

**Authors:** Yong Xie, Yanpan Gao, Licheng Zhang, Yanyu Chen, Wei Ge, Peifu Tang

**Affiliations:** ^1^ Department of Orthopedics Chinese PLA General Hospital Beijing China; ^2^ State Key Laboratory of Medical Molecular Biology Department of Immunology Institute of Basic Medical Sciences Chinese Academy of Medical Sciences School of Basic Medicine Peking Union Medical College Beijing China

**Keywords:** bone remodeling, exosome, osteoblasts, osteoclasts, osteoporosis

## Abstract

Exosomes are secreted into the blood by various types of cells. These extracellular vesicles are involved in the contribution of exosomal proteins to osteoblastic or osteoclastic regulatory networks during the failure of bone remodeling, which results in age‐related bone loss. However, the molecular changes in serum‐derived exosomes (SDEs) from aged patients with low bone density and their functions in bone remodeling remain to be fully elucidated. We present a quantitative proteomics analysis of exosomes purified from the serum of the elderly patients with osteoporosis/osteopenia and normal volunteers; these data are available via Proteome Xchange with the identifier PXD006463. Overall, 1,371 proteins were identified with an overlap of 1,160 Gene IDs among the ExoCarta proteins. Bioinformatics analysis and in vitro studies suggested that protein changes in SDEs of osteoporosis patients are not only involved in suppressing the integrin‐mediated mechanosensation and activation of osteoblastic cells, but also trigger the differentiation and resorption of osteoclasts. In contrast, the main changes in SDEs of osteopenia patients facilitated both activation of osteoclasts and formation of new bone mass, which could result in a compensatory elevation in bone remodeling. While the SDEs from aged normal volunteers might play a protective role in bone health through facilitating adhesion of bone cells and suppressing aging‐associated oxidative stress. This information will be helpful in elucidating the pathophysiological functions of SDEs and aid in the development of senile osteoporosis diagnostics and therapeutics.

## 1 INTRODUCTION

Osteoporosis and osteopenia (low bone mass) are associated with a high risk of fractures, with approximately 25,000 osteoporotic fractures occurring daily, an incidence that is greater than the combined incidence of heart attacks and strokes worldwide. Thus, osteoporosis and osteopenia represent an important global public health issue that is associated with a persistent decrease in the quality of life of affected individuals, especially in the elderly (Barker et al., 2016). These bone disorders are predominantly caused by the failure of bone remodeling, which involves renewal of aged bone and repair of skeletal microdamage through processes that include enhanced osteoclast activity or decreased bone formation from osteoblast lineage cells (Henriksen, Karsdal & Martin, 2014).

Normal bone remodeling is activated by osteoclasts that are unique in their function of bone resorption and generates identifiable scalloped lacunae, followed by a constructive process in which new bone is generated by osteoblasts (Henriksen et al., 2014). The coordinated regulation of these important cell types is critical for maintaining physiological bone remodeling, which is tightly controlled by physical cell–cell interactions, secretory signals, and the endocrine system (Sims & Walsh, 2012). Osteoclast activation occurs after binding of receptor activator of nuclear factor κB (RANKL) to its receptor RANK, which is expressed in the membrane of osteoclast precursors. The signals that positively regulate osteoblast differentiation include members of the integrins, Wnts, and transforming growth factor beta (TGFβ)(Matsuo, 2009). Additionally, recent studies have revealed that various key factors involved in bone remodeling are packaged in spherical bilayered membrane vesicles called exosomes. These organelles function as cell–cell communicators by transferring biologically active molecules to adjacent or distant cells (Xie, Chen, Zhang, Ge & Tang, 2017).

Various cell types secrete exosomes, which are enriched with the markers CD63 and ALG‐2‐interacting protein X (ALIX)(Choi et al., 2012). These organelles are formed solely within the endosomal network and released following fusion of multivesicular bodies with the plasma membrane (Yanez‐Mo et al., 2015). With an average diameter of 40–150 nm, exosomes are released into the circulation and transfer the biologically active molecules contained within their lumen to target cells (Li, Kaslan, Lee, Yao & Gao, 2017). Therefore, rather than representing simple cellular debris, exosomes function as extracellular organelles with local or distant roles in intercellular signaling and communication (Hong et al., 2009). Recent reports indicate the involvement of bone‐associated exosomes in regulating bone remodeling (Li et al., 2016), mainly via the transfer of critical molecules required for the regulation of osteoclasts and osteoblasts (Hao et al., 2017). However, the comprehensive changes among the proteins in serum‐derived exosomes (SDEs) of aged patients with osteoporosis or osteopenia and their functions in bone remodeling remain largely unclear (Colombo, Raposo & Thery, 2014).

Here, to determine the biological functions of SDEs in osteoporosis and osteopenia, we compared the proteomic profiles of exosomes purified from the serum of elderly patients with osteoporosis and low bone mass with those of aged and young normal volunteers using a combination of liquid chromatography–tandem mass spectroscopy (LC‐MS/MS) analyses and tandem mass tag (TMT) label‐based quantitation. Furthermore, we analyzed the phenotypes of osteoclast precursor peripheral blood mononuclear cells (PBMCs), RAW 264.7 cells or osteoblast precursor hFOB 1.19 cells, and MC3T3‐E1 cells following differentiation into mature osteoclasts or osteoblasts in the presence or absence of SDEs. Our study provides evidence that an understanding of the specific cargo of SDEs from patients might be useful for the early evaluation of senile osteoporosis and the development of novel diagnostics and therapeutics.

## 2 RESULTS

### 2.1 Efficiency of SDE extraction and quantitative characteristics of protein profiles

**Figure 1:**
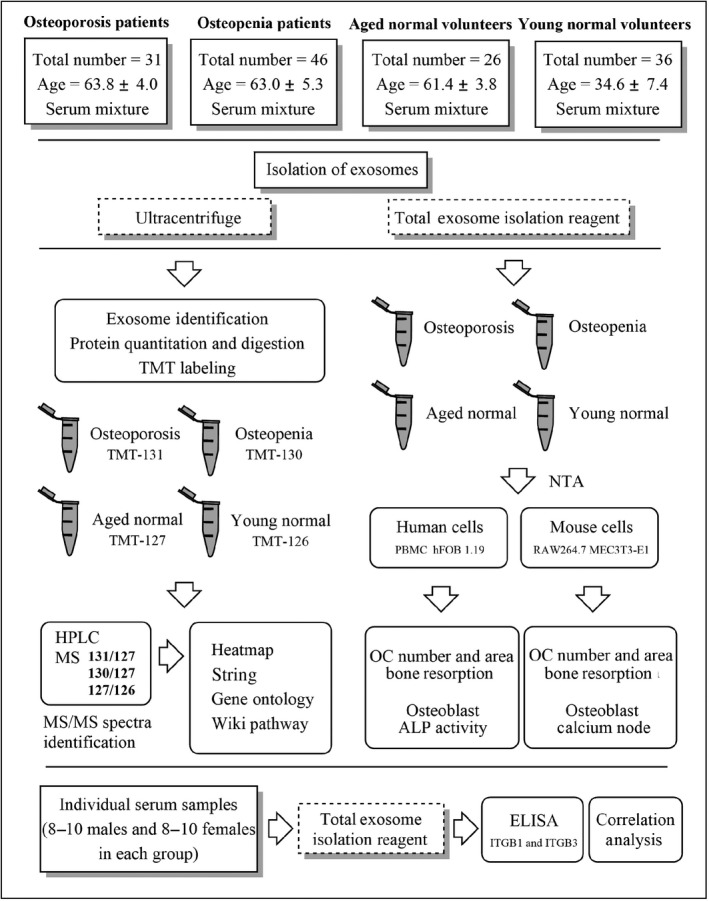
Experimental workflow. “OC” refers to osteoclast, “NTA” refers to nanoparticle tracking analysis

**Figure 2:**
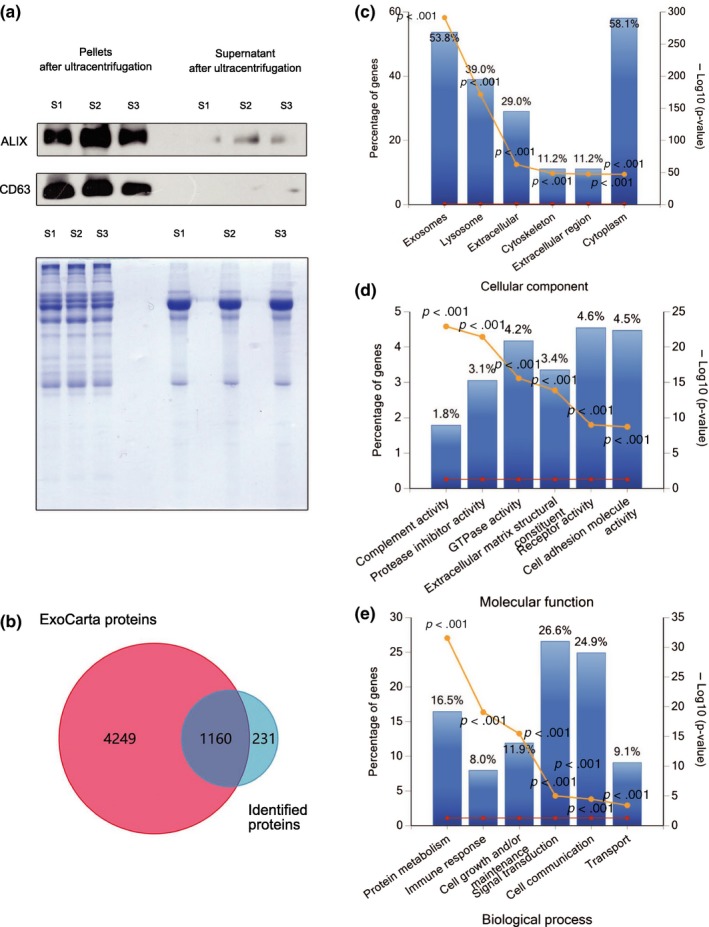
Efficiency of SDE extraction and quantitative characteristics of protein profiles. All identified proteins have been submitted to the ExoCarta database and GO classification system. (a) Western blots showing enrichment of the exosome marker CD63 and ALIX in exosome pellets but not in the supernatants of pooled serum samples after ultracentrifugation. Coomassie brilliant blue staining was used as a control to assess standardized loading. (b) Venn diagram showing the overlap of identified proteins with ExoCarta proteins (5,409; Release date: 29 July 2015). (c–e) The six most enriched categories and the enrichment significance (−log (*p*‐value), *p *<* *.05) of identified proteins in cellular components (c), molecular functions (d), and biological process (e) categories. The percentage of proteins identified in each category is indicated. “S1” refers to pooled serum from Aged normal volunteers, “S2” refers to pooled serum from Aged osteopenia patients, “S3” refers to pooled serum from Aged osteoporosis patients

We compared protein abundances in exosomes purified from serum samples of patients with osteoporosis, osteopenia, and normal volunteers using TMT‐based quantitative MS (Figure 1). To confirm the exosome fractions and validate the exosomal origin of the identified proteins, we compared the expression levels of exosome markers CD63 and ALIX between exosome pellets and supernatants of pooled serum samples after ultracentrifugation (Figure 2a). The raw spectral data were interpreted using Proteome Discoverer 2.1. A total of 1,371 proteins (Score SEQUEST HT >0) were identified (1,350 of 1,371 identifiers from UniProtKB AC/ID were successfully mapped to 1,391 Entrez Gene IDs using the UniProt mapping tool). More than 50% of the identified proteins (679/1,371) exhibited Score SEQUEST HT >10 (Table S1). There was an overlap of 1,160 Entrez Gene IDs in the ExoCarta protein list (http://www.exocarta.org, release date: July 29, 2015) (Figure 2b). Gene Ontology (GO) classification indicated that most of the identified exosomal proteins were derived from cytoplasm and important for molecular functions of receptor activity, cell adhesion molecule activity, and GTPase activity. These proteins were also involved in the biological processes of signal transduction, cell communication, and protein metabolism, which is consistent with the reported functions of exosomes (Figure 2c–e). These data indicated that the purification of exosomes from serum was successful, and the proteins were derived from the SDEs.

The NanoSight reports demonstrated that there were no significant differences between the sizes of SDEs among the different groups. However, there were higher concentrations of SDEs in the Aged normal group compared with those in the Young normal group (Figure S1). Based on the differential expression thresholds (0.5‐fold and twofold change for down‐ and upregulation of expression, respectively), 585 osteoporosis differentially expressed proteins (DEPs, 131/127) and 116 osteopenia DEPs (130/127) were filtered from the results. Among 585 DEPs identified in SDEs isolated from osteoporosis patients relative to those from the aged normal volunteers, 225 proteins were upregulated and 360 proteins were downregulated. In contrast, among the 116 osteopenia DEPs, 110 proteins were upregulated and only six were downregulated. In addition, 59 aged normal DEPs (127/126) were identified. The relative abundances of these proteins are listed in Table S2.

### 2.2 Different expression trends and hub regulators among exosomal proteins in the osteoporosis and osteopenia groups

**Figure 3:**
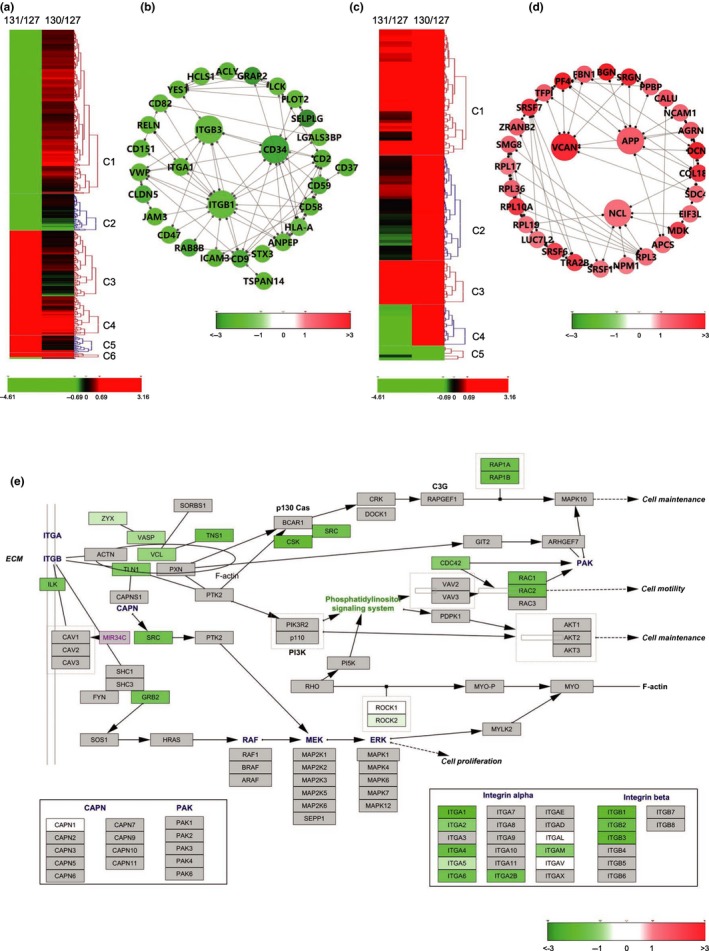
Bioinformatics analysis of DEPs from SDEs based on STRING and Wiki pathway databases. The heatmap was constructed into clusters by Hierarchical Clustering Explorer 3.5 and shows contrasting or similar expression levels of osteoporosis‐associated SDE DEPs compared with those identified from SDEs of osteopenia patients (MS data presented as the ratios to 131/127 and 130/127 were input into Hierarchical Clustering Explorer 3.5 with Ln transformation). The DEPs in the protein–protein interaction networks are shown as nodes (MS data presented as the ratios to 131/127 and 130/127 were matched to STRING networks with log_2_ transformation). Up‐ or downregulation of identified proteins is indicated by colors either in the heatmap or networks (upregulated in red, downregulated in green). All identified proteins were mapped to the relevant Wiki pathway database. Proteins are represented by boxes labeled with the protein name. Relative protein expression level in SDEs of osteoporosis patients is indicated by colors (MS data presented as the ratios to 131/127 and 130/127 were matched to STRING networks with log2 transformation). Proteins in gray were not identified in this study. (a) Heatmap of DEPs of SDEs from patients with osteoporosis (131/127) comparing to those from patients with osteopenia (130/127). (b) Network of overall DEPs in SDEs of patients with osteoporosis. (c) Heatmap of DEPs of SDEs from patients with osteopenia (130/127) comparing to those from patients with osteopenia (131/127). (d) Network of upregulated proteins in SDEs of patients with osteopenia. (e) DEPs of SDEs from osteoporosis patients mapping to the Integrin‐mediated cell adhesion pathway. “C1‐6” refers to cluster 1‐6 in each heatmap

To reveal the differences in expression levels among exosomal proteins in the osteoporosis and osteopenia groups, heatmaps were constructed using Hierarchical Clustering Explorer 3.5 (https://www.cs.umd.edu/hcil/hce). The clusters were identified, and pathway analysis was performed for the proteins in each cluster using the WEB‐based GEne SeT AnaLysis Toolkit (http://bioinfo.vanderbilt.edu/webgestalt/). Information on proteins and pathways in each cluster (Figure 3a,c) is shown in Tables S3 and S4. The expression levels of osteoporosis DEPs (131/127) were significantly lower than those of SDEs from the osteopenia group (130/127) in Cluster 1 (Figure 3a). The mapped results indicated that the osteoporosis DEPs enriched in Cluster 1 were predominantly involved in focal adhesion, G protein signaling, and regulation of calcium and actin cytoskeleton pathways (Table S3), which are implicated in mechanosensation and signal transduction of cell regulation. However, in Cluster 2 (Figure 3a), the decreasing trends in the expression of identified proteins involved in TGF‐β signaling pathways (Table S3) were similar in the osteopenia and osteoporosis groups, although the levels were relatively lower in the osteoporosis group. In contrast, there was no marked difference in the upregulation of identified osteopenia DEPs compared with the expression levels of osteoporosis DEPs in Clusters 3 and 4 (Figure 3a). Additional pathway analysis revealed that these osteoporosis DEPs were most highly enriched in the translation factors and selenium micronutrient network categories (Table S3). However, the increasing trends of expression levels of proteins identified from the osteoporosis group were similar to the osteopenia DEPs in Clusters 1 and 3 (Figure 3c). The mapped results indicated that these osteopenia DEPs were predominantly involved in the cytoplasmic ribosomal protein pathways (Table S4), which are implicated in cell differentiation and highly dynamic metabolic processes. These findings indicated that the specific contents of SDEs perform multiple functions in bone remodeling processes according to their origin and bone health status.

To reveal the potential interactions among the DEPs, comprehensive interaction networks were constructed using the web‐based STRING tools (http://string-db.org). In the network of the osteoporosis DEPs (Figure 3b), the downregulated proteins ITGβ_1_, ITGβ_3_, and hematopoietic progenitor cell antigen CD34 (CD34) represented hubs at which the protein interactions converged. These proteins potentially regulate more than six of the DEPs, most of which are involved in cytoskeletal organization and integrin signaling in cellular adhesion and mechanosensation. The upregulated proteins from SDEs of osteopenia (Figure 3d), such as amyloid precursor protein (APP), NCL, and VCAN, interact with proteins implicated in cellular adhesion and formation of osteoclasts. The networks were visualized using Cytoscape software to clarify the potential relationships between the proteins.

### 2.3 Bioinformatic pathway analysis and GO classification of DEPs

To provide insights into the biological pathways associated with all the DEPs, the WEB‐based GEne SeT AnaLysis Toolkit (http://bioinfo.vanderbilt.edu/webgestalt/) was employed to map the gene symbol of the DEPs to the Wiki pathway database (mapped gene numbers >2; Table S5). Among the osteoporosis DEPs, the upregulated proteins were predominantly enriched in pathways associated with cytoplasmic ribosomal proteins (Table S5A), which are associated with the enhancement of protein metabolism and inflammatory responses. In contrast, 199 of 257 exosomal DEPs derived from plasma membrane were downregulated in the Aged osteoporosis group (Table S3). These DEPs were mapped predominantly to integrin‐mediated cell adhesion, focal adhesion, and G protein signaling pathways, which are important in cell adhesion, mechanosensation, and activation of osteoblasts (Table S5B). In addition, pathway enrichment analysis indicated that several aged normal proteins play pivotal roles in the regulation of bone remodeling by suppressing selenium‐associated oxidative stress, such as intercellular adhesion molecule 1 (ICAM1), serum amyloid A‐1 protein (SAA1), and peroxiredoxin 2 (PRDX2) (Table S5D). To gain further insights, we performed analysis using Cytoscape (3.2.1) software based on the Wiki pathway database. As shown in Figure 3e and Figure S2, all affected proteins were mapped and represented with the same color series with different saturations according to their expression level. Many of downregulated osteoporosis DEPs are known to be involved in pathways associated with integrin‐mediated adhesion and mechanosensation. Among the proteins related to the integrin‐mediated cell adhesion pathway, proteins such as integrin receptors α_1_, β_1_, and β_3_ were strongly downregulated (relative abundance of proteins as ratio of 131/127 < 0.5) in the SDEs of patients with osteoporosis; the downstream of integrin‐linked protein kinase (ILK) and proto‐oncogene tyrosine‐protein kinase Src (SRC) were also downregulated. These results suggested that exosomes in the serum of osteoporosis patients might not be conducive to bone mineralization of osteoblasts in terms of the functional decline of components of the P13K/AKT pathways (Figure 3e). Thus, our data showed that the surface proteins on SDEs were predominantly downregulated in the Aged osteoporosis group, leading to downregulation of osteoclast–osteoblast adhesion, as well as inhibition of mechanosensation of osteoblasts and subsequent mineralization.

In contrast, in the SDEs of osteopenia patients, the DEPs involved in the integrin‐mediated pathway and focal adhesion (Figure S2A) were slightly upregulated (relative abundance of proteins as ratio of 130/127 > 1 but <2). Among the affected proteins involved in the TGF‐β pathways, TGF‐β_1_, LTBP1, and STAT were downregulated in the SDEs of patients with osteoporosis (Figure S2B). These proteins are responsible for coupling bone resorption with bone formation through the SMAD family of signal transduction proteins. The biogenesis of ribosomes is a highly complex and energy‐consuming process that is initiated in the nucleolus (Goudarzi & Lindstrom, 2016), and cytoplasmic ribosomal proteins are upregulated by RANKL‐induced differentiation of osteoclasts. The pathway analysis showed a significant increase in ribosomal proteins (Figure S5A). Guanine nucleotide‐binding proteins (G proteins) principally downregulated in SDEs of osteoporosis patients (Figure S3B) were found to be involved in skeletal system development. Guanine nucleotide‐binding protein G(s) subunit alpha isoforms short (GNAS) is important for osteoblast formation (Hsiao, Millard & Nissenson, 2016), while Kras, a downstream protein of GNAQ, is required for the control of local recruitment of osteoclasts. These findings suggested that the roles of the TGF‐β pathways and G proteins in SDEs of osteoporosis patients are associated with the control of mechanosensation and activation of bone remodeling.

To identify the bone‐related physiological processes implicated by the exosomal proteins, we next clustered overall DEPs (Score SEQUEST HT > 0) into GO categories using bioinformatic tools. In terms of cellular components (Figure S4A), the upregulated osteoporosis DEPs were predominantly enriched in the cytoplasm category, while the downregulated proteins were most likely to be derived from the plasma membrane (Figure S4D). The upregulated osteoporosis DEPs were involved in molecular functions such as structural constituents of ribosomes, protease inhibitor, and chaperone activity (Figure S4B), which are important for the biological processes of protein metabolism and cell growth and/or maintenance (Figure S4C). In contrast, proteins related to signal transduction, cell communication, and transport were downregulated (Figure S4F) and were involved in the molecular functions GTPase activity, receptor activity, and cell adhesion molecule activity (Figure S4E). The DEPs in the osteopenia group had varied characteristics in terms of GO enrichment (Figure S5A–C). The altered proteins from SDEs of the osteopenia group showed a significant enrichment in nucleolus categories. Molecular function analysis revealed that these proteins were mainly responsible for structural constituents of ribosomes and the extracellular matrix and RNA binding, which are important for protein metabolism and cell growth and/or maintenance. These DEPs in the Aged normal group were found to be important for the biological processes of immune responses and cell adhesion, which are predominantly responsible for aging‐related inflammation (Figure S5D–F).

### 2.4 Effects of SDEs on osteoclast differentiation and bone resorption

**Figure 4:**
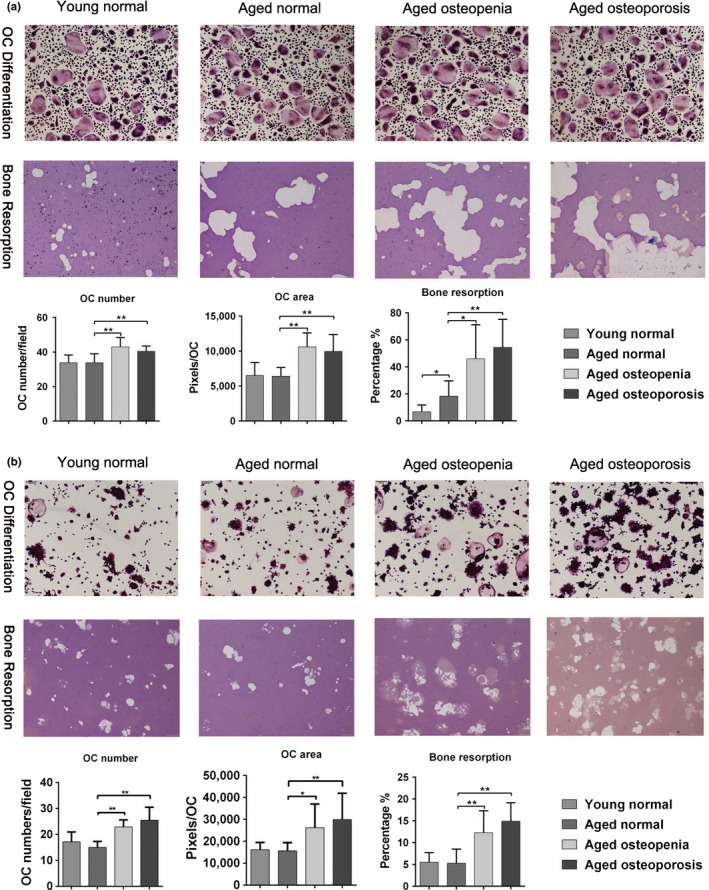
SDEs regulate osteoclast differentiation and bone resorption. (a) The number and area of rearrangement of TRAP‐positive multinucleated osteoclasts cultured with SDEs from osteoporosis or osteopenia patients were significantly higher than that of TRAP‐positive multinucleated osteoclasts cultured with SDEs from aged normal volunteers. Moreover, the bone resorption of osteoclasts cultured with SDEs from osteoporosis or osteopenia patients was significantly greater than the resorption of osteoclasts cultured with aged normal volunteers. In addition, the bone resorption of osteoclasts cultured with SDEs from aged normal volunteers was significantly greater than the resorption of osteoclasts cultured with SDEs from young normal volunteers. However, there was no significant difference in the number and area of rearrangement of between the TRAP‐positive osteoclasts treated with either SDEs from young or aged normal volunteers. (b) RAW264.7 cells cultured with RANKL had a similar effect on osteoclast differentiation and bone resorption compared with the effects of SDEs from different groups; however, the bone resorption of osteoclasts cultured with SDEs from aged normal volunteers was no difference comparing to the resorption of osteoclasts cultured with SDEs from young normal volunteers. Representative photographs are shown in the up panel. Quantification of cells is shown in the down panel. All values are representative of at least two independent experiments with similar results and are displayed as mean ± SD. **p < .01, *p < .05. “OC” refers to osteoclast

We used human PBMCs derived osteoclasts as a model. The number of TRAP‐positive multinucleated osteoclasts cultured with SDEs from osteoporosis or osteopenia patients was significantly higher than the number of osteoclasts cultured with SDEs from aged normal volunteers. Also, the area of rearrangement of TRAP‐positive osteoclasts cultured with SDEs from osteoporosis or osteopenia patients was significantly larger than that of osteoclasts cultured with SDEs from aged normal volunteers. However, there were no significant differences in the number and area of osteoclasts treated with either SDEs from young volunteers or SDEs from elderly normal volunteers. In addition, the bone resorption of osteoclasts cultured with SDEs from osteoporosis or osteopenia patients was significantly stronger than the resorption of osteoclasts cultured with SDEs from aged normal volunteers. We also found that SDEs derived from the elderly normal group had a slightly stronger effect on osteoclast resorption ability than the SDEs derived from younger volunteers. However, the promotion of osteoclast bone resorption by SDEs in the elderly normal group was much weaker than that induced by SDEs in the osteoporosis or osteopenia groups (Figure 4a). RAW264.7 cells cultured with RANKL had a similar effect on osteoclast differentiation and bone resorption compared with the effects of SDEs from different groups (Figure 4b). Taken together, our data suggested that SDEs from both osteoporosis and osteopenia patients promote osteoclast formation in vitro.

### 2.5 Effects of SDEs on osteoblastic bone formation

**Figure 5:**
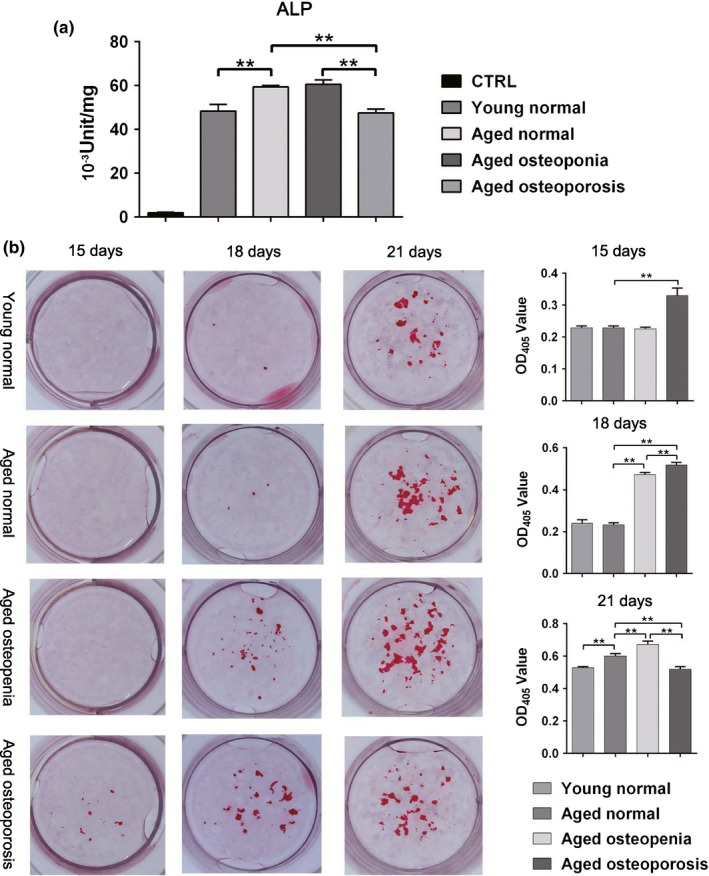
SDEs regulate bone formation by osteoblasts. (a) In the human osteoblastic cell line Hob (hFOB 1.19) cultured on day 21, the alkaline phosphatase (ALP) levels confirmed that higher levels of mineralization of cells cultured with SDEs from osteopenia patients than that of cells cultured with SDEs from aged normal volunteers. However, the level of mineralization in hFOB 1.19 cells cultured with SDEs from osteoporosis patients was significantly lower than that of cells cultured with SDEs from aged normal volunteers. In addition, the mineralization level of cells cultured with SDEs from aged normal was higher than that of cells cultured with SDEs from young normal volunteers. (b) At 15 days after induction of MC3T3‐E1 cells by osteogenic media containing vitamin C and β‐glycerol phosphate, mineralization nodules were first observed in the group cultured in the presence of SDEs from osteoporosis patients. Three days later, mineralization nodules were observed in all the groups, and the OD values of Alizarin Red staining in the Aged osteoporosis and osteopenia groups were higher than those of the Aged normal group. In the later stages of matrix mineralization on day 21, the OD value in the Aged osteoporosis group was lower than those in both the Aged normal and osteopenia groups. Moreover, the OD value in the Aged osteopenia group was higher than that in the Aged normal group. The OD value in the Young normal group was lower than that in the Aged normal group. The OD values of Alizarin Red staining in all these four groups were in accordance with the ALP levels detected in human osteoblastic cells. Representative images are shown in the left panel. Quantification of cells is shown in the right panel. All values are representative of at least two independent experiments with similar results and are displayed as mean ± *SD*. **p < .01, *p < .05

The alkaline phosphatase (ALP) levels of human osteoblastic cell line hFOB 1.19 cultured for 21 days with SDEs from osteopenia patients were higher than those in cells cultured with SDEs from aged normal volunteers. However, the ALP level in hFOB 1.19 cells cultured with SDEs from osteoporosis patients was significantly lower than that of cells cultured with SDEs from aged normal volunteers. In addition, the ALP levels of hFOB 1.19 cells cultured with SDEs from aged normal were higher than those in cells cultured with SDEs from young normal volunteers (Figure 5a).

At 15 days after induction of MC3T3‐E1 cells by osteogenic media containing vitamin C and β‐glycerol phosphate, mineralization nodules were first observed in the group cultured in the presence of SDEs from osteoporosis patients. Three days later, mineralization nodules were observed in all the groups, and the OD values of alizarin red staining in the Aged osteoporosis and osteopenia groups were higher than those of the Aged normal group. In the later stages of matrix mineralization on day 21, the OD value in the Aged osteoporosis group was lower than those in both the Aged normal and osteopenia groups. Moreover, the OD value in the Aged osteopenia group was higher than that in the Aged normal group. The OD value in the Young normal group was lower than that in the Aged normal group. The OD values of Alizarin Red staining in all these four groups were in accordance with the ALP levels detected in human osteoblastic cells (Figure 5b). Taken together, our data suggested that SDEs from osteopenia patients and aged normal volunteers promote osteoblast formation in vitro, while SDEs from osteoporosis patients inhibit osteoblast formation in osteogenesis.

### 2.6 Verification of protein expression levels by representative MS/MS spectral identification and ELISA

**Figure 6:**
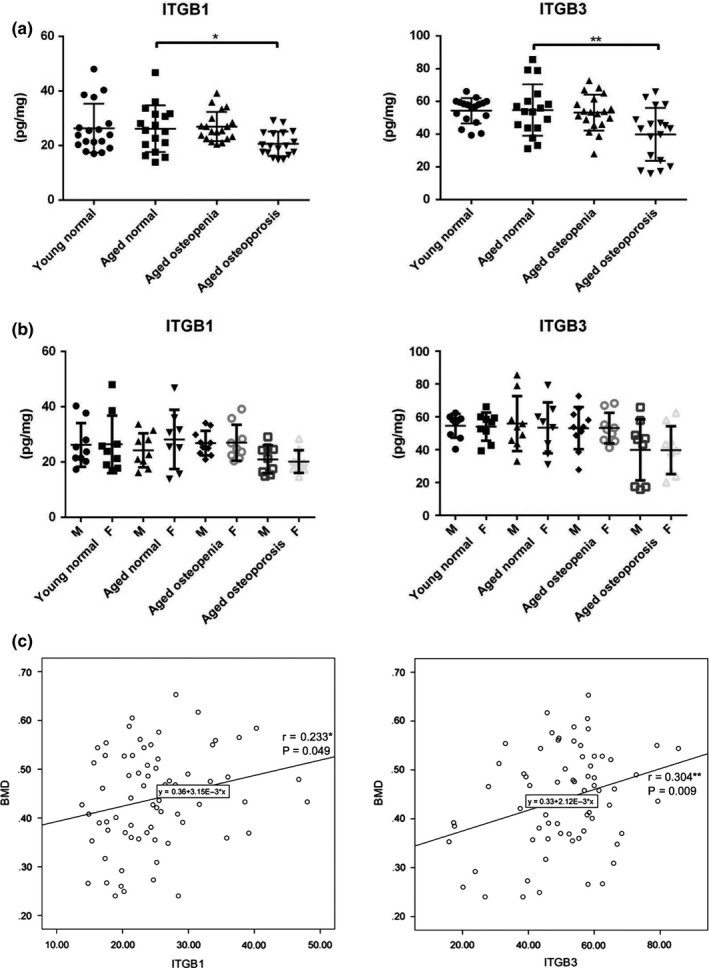
Verification of protein expression levels by ELISA and correlation analysis between the changes in exosomal proteins and BMD. To validate the MS results, (a) the SDEs of 72 serum samples were subjected to ELISA using antibodies for specific detection of integrin β_1_ (ITGB1) and integrin β_3_ (ITGB3), individually. The fluctuation in the levels of these proteins was consistent with the proteomics data. (b) The data also demonstrated that there was no significant difference between the male and female patients in each group. (c) Analysis of the correlation between the expression level of ITGB1 and ITGB3 in SDEs and BMD. “BMD” refers to bone mass density

To validate the MS results, the identified SDE proteins were subjected to representative mass spectral analysis. According to the bioinformatics analysis, we chose four proteins (integrin β1, integrin β3, TGFβ1, and APP) to process representative MS/MS spectral identification (Figure S6A–D). In addition, 72 serum samples (8–10 males and 8–10 females in each group) were analyzed to ELISA using antibodies for the specific detection of integrin β1 and integrin β3, individually (Figure 6a). The fluctuation in the levels of these proteins was consistent with the proteomics data. The ELISA data also demonstrated that there were no significant differences in the levels of these proteins between the male and female patients in each group (Figure 6b). Statistical analysis of the results demonstrated that the expression of exosomal integrin β_1_ and β_3_ was positively correlated with BMD among groups (Figure 6c).

## 3 DISCUSSION

Previous studies have demonstrated the multiple roles of bone‐derived exosomes in bone remodeling; however, none have been reported describing proteomics analysis of the differences between SDEs from aged patients with low bone density and normal volunteers. In the present study, we discovered that the SDEs from osteoporosis patients inhibited osteoblastic bone matrix mineralization and promoted osteoclast differentiation. In contrast, SDEs from osteopenia patients enhanced both osteoblast function and osteoclast activation, leading to a compensatory increase in bone remodeling. A comprehensive analysis of the changes in proteins in these exosomes was conducted by TMT‐based MS, which has the advantages of maximum protein coverage and precise quantification. The DEPs identified were involved in different processes and functions intrinsic to bone, including mechanosensation, inflammation, and cell senescence, which are the apparent protagonists in bone remodeling.

### 3.1 Exosomal integrin‐related proteins which involved in mechanosensation and activation of bone‐related pathways may play pivotal roles in bone remodeling

We revealed the decrease in osteoblastic bone formation by treatment with SDEs from patients with osteoporosis. Furthermore, our analysis showed that proteins implicated in the processes of integrin‐mediated adhesion and mechanosensation were downregulated in the SDEs of patients with osteoporosis. Exosome binding by recipient cells is likely to be determined by a repertoire of integrins, which dictate exosome adhesion to specific cell types and ECM molecules (Hoshino et al., 2015). Among the DEPs of SDEs of osteoporosis patients, the β_1_ and β_3_ integrins and CD34 were representatives of the downregulated proteins. Previous studies indicated that integrin α_v_β_3_ is essential for mechanosensation and osteoblastic bone cells express integrin receptors α_v_, β_1_, and β_3_ (Haugh, Vaughan & McNamara, 2015). Another study demonstrated that mechanical tensile strain using a four‐point bending device promoted integrin β_1_‐mediated Wnt/β‐catenin nuclear translocation, which induced the expression of an osteoblastic transcriptional factor, osterix (Kobayashi, Uehara, Udagawa & Takahashi, 2016). CD34 mediates the attachment of stem cells to the bone marrow ECM or directly to stromal cells; circulating CD34^+^ cells are capable of differentiating into osteoblasts (Kuroda et al., 2014). Furthermore, the integrin β_1_/Shc association leads to the activation of extracellular signal‐regulated kinase (ERK), which is critical for shear induction of bone formation‐related genes in osteoblast‐like cells (Lee et al., 2008). Integrin‐linked protein kinase (ILK) might regulate ERK signaling through indirect interaction. Hoshino et al. (2015) demonstrated that, in addition to adhesive properties, exosomal integrin uptake can activate SRC in specific resident cells. SRC is an upstream signaling partner of ERK and regulates SMAD nuclear translation through activation of osterix and results in osteoblastic bone mineralization (Choi et al., 2015). These integrin‐related proteins were largely downregulated in SDEs of osteoporosis patients in this study. Additionally, statistical analysis of our results demonstrated that the expression of exosomal integrin β_1_ and β_3_ is positively correlated with BMD among the groups (Figure 6c), indicating that β_1_ and β_3_ integrins are potential biomarkers of bone status and osteoporotic risks. In accordance with the results of our bioinformatics analysis, exosomal β_1_ and β_3_ integrins were both downregulated when the mean *T*‐score decreased from −1.70 to −3.41 among the groups. Therefore, we propose that reductions in integrin‐mediated activation of ERK signaling, as well as their downstream signaling cascade, in SDEs from osteoporosis patients might impair mechanosensation responses to mechanical stimuli and activation of osteoblastic bone cells throughout the body. Accordingly, detection of the expression level of these integrins in serum exosomes would be beneficial in predicting osteoporotic risks and analyzing the results of therapy. High expression of β_1_ and β_3_ integrins in serum exosomes implies that the bone system is undergoing a process of compensatory bone reconstruction, while downregulation of these integrins indicates that the failure of bone remodeling is advanced.

In addition, we found that SDEs of osteoporosis patients with uniformly low levels of TGF‐β cascade‐associated proteins promoted the differentiation of osteoclasts. TGF‐β is one of the key cytokines responsible for coupling bone resorption with bone formation, largely by recruitment of MSCs to bone‐resorptive sites. Signaling by TGF‐β in MSCs occurs through the SMAD family of signal transduction proteins. The gradient of TGF‐β created during osteoclast bone resorption can limit further osteoclast activity (Crane & Cao, 2014). LTBP1 is involved in the assembly, secretion, and targeting of TGF‐β_1_ to sites at which it is stored and/or activated. In addition, LOF mutations of STAT3 decrease the osteogenic response following mechanical loading (Li, 2013). Thus, downregulated TGF‐β signaling may lead to loss of skeletal integrity via enhancement of osteoclast bone resorption and interrupted bone formation. However, numbers of cytoplasmic ribosomal proteins were increased in SDEs of osteoporosis patients. Ribosomes are cellular machines that are essential for protein synthesis (Trainor & Merrill, 2014). The biogenesis of ribosomes is a highly complex and energy‐consuming process that is initiated in the nucleolus, and cytoplasmic ribosomal proteins are upregulated by RANKL‐induced differentiation of osteoclasts (Day et al., 2004). From this, we propose that SDEs of osteoporosis patients not only suppress the mechanosensation response of target cells, but also trigger signaling pathways for elevation of osteoclastic differentiation and exacerbate abnormal bone remodeling.

### 3.2 Exosomes in the circulation of patients with osteopenia could result in a compensatory elevation of bone remodeling

Our in vitro studies show that SDEs of patients with osteopenia facilitate the adhesion and activation of osteoclasts and formation of new bone mass. In accordance with these results, our analysis showed several hub proteins implicated in the regulation of osteoblasts and osteoclasts. The most important proteins that were found to be upregulated in SDEs of osteopenia patients were amyloid precursor protein (APP), nucleolin (NCL), and members of the cytoplasmic ribosomal proteins. APP and NCL have been reported to be involved in cellular adhesion and survival of osteoclasts. The abnormal APP/Aβ deposition in osteoporotic bone seems to be associated with potent enhancement of osteoclast differentiation and activation, suggesting an important role for Aβ in the pathogenesis of osteoporosis. These signaling cascades may initiate nuclear factor of activated T cells c1 (NFATc1) translocation into the nucleus, eventually inducing osteoclast‐specific gene transcription to allow osteoclast differentiation and activation (Li, Liu, Zhang & Rong, 2014). All osteoclast nuclei are transcriptionally active, and the functional role is reflected by the expression of regulatory proteins that support ribosomal RNA synthesis, such as NCL, which is thought to play a role in pre‐rRNA transcription and ribosome assembly (Saltman et al., 2005). Therefore, the upregulated cytoplasmic ribosomal proteins in SDEs of both osteoporosis and osteopenia patients may function as positive feedback factors in the activation of osteoclasts.

In contrast to SDEs of osteoporosis patients, those of osteopenia patients promote osteoblastic bone formation. Our analysis indicates that versican core protein (VCAN) and connective tissue growth factor (CTGF) are upregulated in SDEs of osteopenia patients and act as hubs in regulating the function of osteoblasts. VCAN is an ECM component that plays an essential role in transformed cell behavior. Increases in VCAN have been confirmed in human MSCs undergoing osteoblast differentiation (Foster et al., 2005). CTGF serves as an adhesive substrate for promoting cell spreading via cytoskeletal reorganization as well as enhancing osteoblast adhesion via integrin α_v_β_1_. Consistently, integrin‐mechanosensation‐related proteins were slightly upregulated (relative abundance of proteins as ratio of 130/127 > 1 but <2). Additionally, fibrillin‐1 (FBN1) was constitutively upregulated in SDEs of osteopenia patients. FBN1 mediates cell adhesion via its binding to cell surface receptors integrin α_v_β_1_ and selectively blunts expression of osterix, which is a transcriptional regulator of osteoblast maturation (Nistala et al., 2010). In terms of the adhesion and activation of osteoclasts that are accompanied by elevated bone formation, we believe that these DEPs from exosomes in the circulation of patients with osteopenia are involved in a compensated elevation of bone remodeling, which could result in a relatively low level of bone destruction and bone mass reduction.

### 3.3 SDEs from aged normal volunteers involved in cell adhesion and oxidative stress might function as protective regulators of bone health and the aging process

The aging skeletal system undergoes a progressive decline in the renewal of bone tissue; therefore, we investigated the changes in protein expression of SDEs from elderly volunteers without bone loss compared with those from young normal volunteers. The 59 aged normal DEPs were found to be important for the biological processes of immune responses, which are predominantly responsible for alleviation of aging‐related inflammation (Figure S5F). Pathway enrichment analysis indicated that several selenium‐associated proteins play pivotal roles in the regulation of bone remodeling by inhibiting oxidative stress. Selenium is beneficial to total BMD (Beukhof et al., 2016). Selenite protects MSCs against H_2_O_2_‐induced inhibition of osteoblast differentiation through suppressing oxidative stress and ERK activation (Liu, Bian, Liu & Huang, 2012). Furthermore, selenium deficiency is detrimental to bone microarchitecture by increasing bone resorption, possibly through decreasing antioxidative potential (Cao, Gregoire & Zeng, 2012). The representative proteins, such as ICAM1 and SAA1, were upregulated in the SDEs of elderly volunteers recruited in this study, while PRDX2 was downregulated. ICAM1 provides high‐affinity adhesion between osteoblast and osteoclast precursors, thereby facilitating osteoclast activity and inhibiting bone formation (Cheung, Simmons & You, 2012). SAA1, a major acute phase protein, stimulates MMP9 production for bone resorption (Paret, Schon, Szponar & Kovacs, 2010). In contrast, peroxiredoxin 2 (PRDX2), a member of the antioxidant enzyme family, is protective against oxidative damage during osteoclastogenesis (Park et al., 2015).

As shown in the heatmap (Figure S7C), 33 overlapping DEPs were identified in the Aged normal and Aged osteoporosis groups. Of these, 27 were highly expressed in the Aged normal group but were expressed at low levels in the Aged osteoporosis group (Table S2). Among these proteins, we found that CD44, ICAM, and ITGA1 play pivotal roles in resisting age‐related bone loss via mediation of cell–cell and cell–matrix adhesion (Zu, Liang, Du, Zhou & Yang, 2015). However, owing to the small number of overlapping DEPs (both <10%; 33 of 574 in the osteoporosis group and nine of 112 in the osteopenia group. Figure S7A), this effect of aged normal SDEs was weak compared with the robust alteration of exosomal proteins in SDEs from elderly patients with low bone density. In addition, we revealed that the SDEs in the Aged normal group promoted osteoblastic bone formation compared with the effects of SDEs in the Young normal group. Moreover, the bone resorption areas observed in Aged normal group were larger than those in the Young normal group. However, these effects were far weaker than those of SDEs from patients with bone loss. Furthermore, there were higher concentrations of SDEs in the Aged normal group compared with those in the young group (Figure S1), which might be one reason for the positive effects of these SDEs on osteoclast differentiation and bone formation. Therefore, we infer that the exosomes in the circulation of elderly normal people function as protective regulators of bone health by maintaining the homeostasis of bone health via facilitating high‐affinity adhesion and suppressing selenium‐associated oxidative stress. Thus, our studies indicate that age might not be the most important factor influencing the failure of bone remodeling regulated by SDEs. The decline in bone quality is more likely to be attributed to the SDEs derived from abnormal bone cells rather than the aging process. Increasing the expression level of protective regulators in SDEs could be beneficial for bone health in aged people.

Taken together, the mechanisms by which the content of exosomes is selected remain unclear, particularly in bone cells. However, the presence of DEPs implicated in different bone remodeling processes suggests that the process by which the exosomal cargo is selected is not random (Meehan & Vella, 2016). According to our findings on the different functions of up‐ and downregulated DEPs in SDEs from patients with bone disorders or elderly people, we speculate that the protein content of exosomes is determined by the stage of progressive failure of bone remodeling. Exosomal proteins might be secreted into the circulation and transported to the target cell responsible for activation of the bone remodeling process. During the process of purification from serum, we co‐isolated a mixed population of exosomes without further demonstration of their intracellular origin. From the data presented, it is not clear which types of bone cells are the main sources of SDEs and whether different subsets of exosomes are more prone to being targeted into the circulation and transported to regions that are distant from the local bone cells. Although the different functions of SDEs need further delineation, detection of the contents of SDEs might provide an early understanding of the progression of the stages of bone destruction. Integrin expression profiles of circulating exosomes isolated from patients with bone loss could be used as prognostic factors to predict changes in bone quality. Hence, upregulation of β_1_ and β_3_ integrins in the exosome for integrin‐mediated mechanosensation might result in transportation to the target cell responsible for activation of the bone remodeling process.

## 4 EXPERIMENTAL PROCEDURES

### 4.1 Patients and serum samples

The patients diagnosed with osteoporosis and osteopenia at the Chinese PLA General Hospital (PLAGH, Beijing, China) were recruited to this study from September 2015 to December 2017. For quantitative proteomics analysis, serum samples from 31 osteoporosis patients, and 46 osteopenia patients (aged 55–84) were allocated to Aged osteoporosis and Aged osteopenia groups. Serum samples obtained from 26 elderly normal volunteers aged 56 to 70 and 36 normal volunteers aged 21 to 49 were allocated to Aged normal and Young normal groups, respectively (information shown in Table S6). For individual validation, 72 additional serum samples (8–10 males and 8–10 females in each group) were collected (information shown in Table S7). All these serum samples were collected at the same time of day and under fasting conditions. All the patients and volunteers involved in the study were without diabetes, thyroid diseases, autoimmune diseases, or tumors and had not received treatment for osteopenia or osteoporosis prior to this study; their information was obtained from the PLAGH. BMD‐based bone assessments were measured by DXA (OsteoSys EXA 3000 Bone Density Device) at the one‐third radius site. In postmenopausal women and men aged ≥50, osteoporosis and osteopenia were diagnosed according to the application of the WHO diagnostic *T*‐score criteria to BMD measurements (with *T*‐scores at −1.0 or above defined as “normal”; between −1.0 and −2.5 defined as “osteopenia”; and at −2.5 or below defined as “osteoporosis”). However, in premenopausal women, and men <50 years of age, the ethnicity or race adjusted *Z*‐scores were recommended by the International Society for Clinical Densitometry (ISCD) instead of *T*‐scores in the diagnostic classification (with *Z*‐scores of −2.0 or lower defined as either “low bone mineral density for chronological age” or “below the expected range for age” and those above −2.0 defined as “within the expected range for age”). Informed consent was obtained from all patients and normal volunteers. The study was performed with the approval of the Ethics Committee of the PLAGH.

### 4.2 Isolation of exosomes from human serum

Exosomes were isolated from pooled serum samples belonging to different groups by ultracentrifugation and using the Total Exosome Isolation Reagent as previously reported (Chen et al., 2017). In brief, serum was first diluted with an equal volume of PBS. For ultracentrifugation, the diluted serum was centrifuged at 10,000 × *g* for 30 min at 4°C followed by ultracentrifugation at 110,000 × *g* at 4°C for 90 min using a Beckman Optima L‐100XP Ultracentrifuge. The pellet was washed with 1 ml PBS and then dissolved with 40 μl 8 M urea (Kim, Tan & Lubman, 2015). The protein concentration was measured with a NanoDrop 2000 spectrophotometer (Thermo Scientific). For exosome isolation, 0.2 volumes of the Total Exosome Isolation Reagent were added to diluted serum and vortexed for 30 s prior to incubation at 4°C for 30 min. The sample was then centrifuged at 10,000 × *g* for 10 min at room temperature to pellet the exosomes. For osteoclastogenesis and bone formation assays, the exosome‐enriched pellet was resuspended in PBS at a ratio of 25 μl PBS per 100 μl serum. The exosome suspension was lysed with RIPA lysis buffer, and protein concentration was determined using a BCA protein assay kit.

### 4.3 Bioinformatics analysis

Relative protein abundances are presented as the ratios to TMT‐131/127, 130/127, and 127/126.

The differential expression threshold was defined as a twofold change for both down‐ and upregulation of expression. The mass spectrometry proteomics data have been deposited to the ProteomeXchange Consortium via the PRIDE (Vizcaino et al., 2016) partner repository with the dataset identifier PXD006463. GO analysis was performed with FunRich software (2.1.2) (Pathan et al., 2015). The Entrez Gene IDs retrieved from UniProtKB accession numbers were mapped to cellular components (CC), molecular functions (MF), and biological processes (BP) items using default statistical parameters (threshold: count 2, ease 0.1). The UniProtKB accession numbers were uploaded directly using the WEB‐based GEne SeT AnaLysis Toolkit (http://bioinfo.vanderbilt.edu/webgestalt/) for pathway mapping. Pathways with at least three target genes and a *p*‐value of <.05 were considered to be statistically significant. Enriched pathways were visualized by Cytoscape (3.2.1) software. For Hierarchical clustering analysis, the MS proteomics data presented as the ratios to 131/127 and 130/127 were input into Hierarchical Clustering Explorer 3.5 with log transformation (natural), and a heatmap was then constructed using the complete linkage method. At the beginning of the process, each element is in a cluster of its own. The clusters are then sequentially combined into larger clusters until all elements are combined as a single cluster. In each step, the two clusters separated by the shortest distance are combined (Brian S. Everitt; Sabine Landau; Morven Leese (2001). Cluster Analysis (Fourth ed.). London: Arnold. ISBN 0‐340‐76119‐9.). The STRING (Search Tool for the Retrieval of Interacting Genes/Proteins) database (http://string-db.org) was used for predicting protein networks. All STRING network analyses were performed using UniProt accession numbers as the input and with “Experimental” and “Database” evidence at medium (0.4) confidence level. The networks were downloaded as tab‐delimited text files, and visualized, and reorganized using Cytoscape (3.2.1) software. In addition, the MS proteomics data presented as the ratios to 131/127 and 130/127 were matched to these STRING networks with log_2_ transformation.

### 4.4 Effects of SDEs on osteoclastogenesis and osteoblastic bone formation

SDEs (200 μg) from each of the Young normal, Aged normal, Aged osteopenia, and Aged osteoporosis groups were added to RAW 264.7 cells (1.5 × 10^5^ cells/cm^2^) or PBMCs; each group was prepared in triplicate. The culture medium was refreshed every other day. On day 5, cells were confirmed by TRAP staining and photographed for evaluation of numbers and area rearrangements. SDEs (200 μg) from each of the Young normal, Aged normal, Aged osteopenia, and Aged osteoporosis groups were added to MC3T3‐E1 cells (1.5 × 10^5^ cells/cm^2^) or hFOB 1.19 cell lines; each group was prepared in triplicate. On days 15, 18, and 21, MC3T3‐E1 cells were photographed and confirmed by Alizarin Red staining, and the sample‐bound Alizarin Red staining of each group was solubilized for OD measurements. On day 21, ALP levels of hFOB 1.19 cells were assayed. The supplementary experimental procedures were shown in Appendix S1.

### 4.5 Statistical analysis

Data are expressed as the mean ± *SD* of triplicate experiments, unless otherwise indicated. Statistical differences among groups were analyzed by one‐way analysis of variance with a post hoc test to determine group differences in the study parameters. All statistical analyses were performed with SPSS software (version 24.0 for Windows; Armonk, NY, USA) and Prism software (GraphPad prism for Windows, version 6.01; Nashville, TN, USA). Unless otherwise indicated, data were analyzed using Student's *t* test, and *p *<* *.05 was considered to indicate statistical significance.

## CONFLICT OF INTEREST

The authors report no conflict of interest relevant to this article.

## AUTHOR'S CONTRIBUTIONS

P.T. and W.G. designed the research. Y.X., Y.G., and L.Z. performed the research. Y.X., Y.G., and Y.C. analyzed the data. L.Z. provided serum samples and reagents. Y.X. and Y.G. wrote the manuscript.

## References

[acel12758-bib-0001] Barker, A. L. , McNeil, J. J. , Seeman, E. , Ward, S. A. , Sanders, K. M. , Khosla, S. , … Talevski, J. (2016). A randomised controlled trial of low‐dose aspirin for the prevention of fractures in healthy older people: Protocol for the ASPREE‐Fracture substudy. Injury Prevention: Journal of the International Society for Child and Adolescent Injury Prevention, 22, 297–301. 10.1136/injuryprev-2015-041655 26002770 PMC4879092

[acel12758-bib-0002] Beukhof, C. M. , Medici, M. , van den Beld, A. W. , Hollenbach, B. , Hoeg, A. , Visser, W. E. , … Peeters, R. P. (2016). Selenium status is positively associated with bone mineral density in healthy aging European men. PLoS ONE, 11, e0152748 10.1371/journal.pone.0152748 27055238 PMC4824523

[acel12758-bib-0003] Cao, J. J. , Gregoire, B. R. , & Zeng, H. (2012). Selenium deficiency decreases antioxidative capacity and is detrimental to bone microarchitecture in mice. The Journal of Nutrition, 142, 1526–1531. 10.3945/jn.111.157040 22739365

[acel12758-bib-0004] Chen, Y. , Xie, Y. , Xu, L. , Zhan, S. , Xiao, Y. , Gao, Y. , … Ge, W. (2017). Protein content and functional characteristics of serum‐purified exosomes from patients with colorectal cancer revealed by quantitative proteomics. International Journal of Cancer, 140, 900–913. 10.1002/ijc.30496 27813080

[acel12758-bib-0005] Cheung, W. Y. , Simmons, C. A. , & You, L. (2012). Osteocyte apoptosis regulates osteoclast precursor adhesion via osteocytic IL‐6 secretion and endothelial ICAM‐1 expression. Bone, 50, 104–110. 10.1016/j.bone.2011.09.052 21986000

[acel12758-bib-0006] Choi, Y. H. , Han, Y. , Lee, S. H. , Cheong, H. , Chun, K. H. , Yeo, C. Y. , & Lee, K. Y. (2015). Src enhances osteogenic differentiation through phosphorylation of Osterix. Molecular and Cellular Endocrinology, 407, 85–97. 10.1016/j.mce.2015.03.010 25802190

[acel12758-bib-0007] Choi, D. S. , Yang, J. S. , Choi, E. J. , Jang, S. C. , Park, S. , Kim, O. Y. , … Gho, Y. S. (2012). The protein interaction network of extracellular vesicles derived from human colorectal cancer cells. Journal of Proteome Research, 11, 1144–1151. 10.1021/pr200842h 22149170

[acel12758-bib-0008] Colombo, M. , Raposo, G. , & Thery, C. (2014). Biogenesis, secretion, and intercellular interactions of exosomes and other extracellular vesicles. Annual Review of Cell and Developmental Biology, 30, 255–289. 10.1146/annurev-cellbio-101512-122326 25288114

[acel12758-bib-0009] Crane, J. L. , & Cao, X. (2014). Bone marrow mesenchymal stem cells and TGF‐beta signaling in bone remodeling. The Journal of Clinical Investigation, 124, 466–472. 10.1172/JCI70050 24487640 PMC3904610

[acel12758-bib-0010] Day, C. J. , Kim, M. S. , Stephens, S. R. , Simcock, W. E. , Aitken, C. J. , Nicholson, G. C. , & Morrison, N. A. (2004). Gene array identification of osteoclast genes: Differential inhibition of osteoclastogenesis by cyclosporin A and granulocyte macrophage colony stimulating factor. Journal of Cellular Biochemistry, 91, 303–315. 10.1002/(ISSN)1097-4644 14743390

[acel12758-bib-0011] Foster, L. J. , Zeemann, P. A. , Li, C. , Mann, M. , Jensen, O. N. , Kassem, M. (2005). Differential expression profiling of membrane proteins by quantitative proteomics in a human mesenchymal stem cell line undergoing osteoblast differentiation. Stem Cells (Dayton, Ohio) 23, 1367–1377. 10.1634/stemcells.2004-0372 16210410

[acel12758-bib-0012] Goudarzi, K. M. , & Lindstrom, M. S. (2016). Role of ribosomal protein mutations in tumor development (Review). International Journal of Oncology, 48, 1313–1324. 10.3892/ijo.2016.3387 26892688 PMC4777597

[acel12758-bib-0013] Hao, Z. C. , Lu, J. , Wang, S. Z. , Wu, H. , Zhang, Y. T. , Xu, S. G. (2017). Stem cell‐derived exosomes: A promising strategy for fracture healing. Cell Proliferation 50, e12359. 10.1111/cpr.12359 PMC6529061 28741758

[acel12758-bib-0014] Haugh, M. G. , Vaughan, T. J. , & McNamara, L. M. (2015). The role of integrin alpha(V)beta(3) in osteocyte mechanotransduction. Journal of the Mechanical Behavior of Biomedical Materials, 42, 67–75. 10.1016/j.jmbbm.2014.11.001 25460927

[acel12758-bib-0015] Henriksen, K. , Karsdal, M. A. , & Martin, T. J. (2014). Osteoclast‐derived coupling factors in bone remodeling. Calcified Tissue International, 94, 88–97. 10.1007/s00223-013-9741-7 23700149

[acel12758-bib-0016] Hong, B. S. , Cho, J. H. , Kim, H. , Choi, E. J. , Rho, S. , Kim, J. , … Gho, Y. S. (2009). Colorectal cancer cell‐derived microvesicles are enriched in cell cycle‐related mRNAs that promote proliferation of endothelial cells. BMC Genomics, 10, 556 10.1186/1471-2164-10-556 19930720 PMC2788585

[acel12758-bib-0017] Hoshino, A. , Costa‐Silva, B. , Shen, T. L. , Rodrigues, G. , Hashimoto, A. , Tesic Mark, M. , … Lyden, D. (2015). Tumour exosome integrins determine organotropic metastasis. Nature, 527, 329–335. 10.1038/nature15756 26524530 PMC4788391

[acel12758-bib-0018] Hsiao, E. C. , Millard, S. M. , Nissenson, R. A. (2016). Gs/Gi regulation of bone cell differentiation: Review and insights from engineered receptors. Hormone and Metabolic Research 48, 689–699. 27643449 10.1055/s-0042-116156 PMC13032884

[acel12758-bib-0019] Kim, J. , Tan, Z. , & Lubman, D. M. (2015). Exosome enrichment of human serum using multiple cycles of centrifugation. Electrophoresis, 36, 2017–2026. 10.1002/elps.201500131 26010067 PMC4558199

[acel12758-bib-0020] Kobayashi, Y. , Uehara, S. , Udagawa, N. , & Takahashi, N. (2016). Regulation of bone metabolism by Wnt signals. Journal of Biochemistry, 159, 387–392. 10.1093/jb/mvv124 26711238 PMC4885935

[acel12758-bib-0021] Kuroda, R. , Matsumoto, T. , Kawakami, Y. , Fukui, T. , Mifune, Y. , & Kurosaka, M. (2014). Clinical impact of circulating CD34‐positive cells on bone regeneration and healing. Tissue Engineering Part B, Reviews, 20, 190–199. 10.1089/ten.teb.2013.0511 24372338 PMC4030654

[acel12758-bib-0022] Lee, D. Y. , Yeh, C. R. , Chang, S. F. , Lee, P. L. , Chien, S. , Cheng, C. K. , & Chiu, J. J. (2008). Integrin‐mediated expression of bone formation‐related genes in osteoblast‐like cells in response to fluid shear stress: Roles of extracellular matrix, Shc, and mitogen‐activated protein kinase. Journal of Bone and Mineral Research: The Official Journal of the American Society for Bone and Mineral Research, 23, 1140–1149. 10.1359/jbmr.080302 18333755

[acel12758-bib-0023] Li, J. (2013). JAK‐STAT and bone metabolism. JAK‐STAT, 2, e23930 10.4161/jkst.23930 24069548 PMC3772100

[acel12758-bib-0024] Li, P. , Kaslan, M. , Lee, S. H. , Yao, J. , & Gao, Z. (2017). Progress in exosome isolation techniques. Theranostics, 7, 789–804. 10.7150/thno.18133 28255367 PMC5327650

[acel12758-bib-0025] Li, D. , Liu, J. , Guo, B. , Liang, C. , Dang, L. , Lu, C. , … Zhang, G. (2016). Osteoclast‐derived exosomal miR‐214‐3p inhibits osteoblastic bone formation. Nature Communications, 7, 10872 10.1038/ncomms10872 PMC4786676 26947250

[acel12758-bib-0026] Li, S. , Liu, B. , Zhang, L. , & Rong, L. (2014). Amyloid beta peptide is elevated in osteoporotic bone tissues and enhances osteoclast function. Bone, 61, 164–175. 10.1016/j.bone.2014.01.010 24473375

[acel12758-bib-0027] Liu, H. , Bian, W. , Liu, S. , & Huang, K. (2012). Selenium protects bone marrow stromal cells against hydrogen peroxide‐induced inhibition of osteoblastic differentiation by suppressing oxidative stress and ERK signaling pathway. Biological Trace Element Research, 150, 441–450. 10.1007/s12011-012-9488-4 22890880

[acel12758-bib-0028] Matsuo, K. (2009). Cross‐talk among bone cells. Current Opinion in Nephrology and Hypertension, 18, 292–297. 10.1097/MNH.0b013e32832b75f1 19395964

[acel12758-bib-0029] Meehan, K. , & Vella, L. J. (2016). The contribution of tumour‐derived exosomes to the hallmarks of cancer. Critical Reviews in Clinical Laboratory Sciences, 53, 121–131. 10.3109/10408363.2015.1092496 26479834

[acel12758-bib-0030] Nistala, H. , Lee‐Arteaga, S. , Smaldone, S. , Siciliano, G. , Carta, L. , Ono, R. N. , … Ramirez, F. (2010). Fibrillin‐1 and ‐2 differentially modulate endogenous TGF‐beta and BMP bioavailability during bone formation. The Journal of Cell Biology, 190, 1107–1121. 10.1083/jcb.201003089 20855508 PMC3101602

[acel12758-bib-0031] Paret, C. , Schon, Z. , Szponar, A. , & Kovacs, G. (2010). Inflammatory protein serum amyloid A1 marks a subset of conventional renal cell carcinomas with fatal outcome. European Urology, 57, 859–866. 10.1016/j.eururo.2009.08.014 19747761

[acel12758-bib-0032] Park, H. , Noh, A. L. , Kang, J. H. , Sim, J. S. , Lee, D. S. , & Yim, M. (2015). Peroxiredoxin II negatively regulates lipopolysaccharide‐induced osteoclast formation and bone loss via JNK and STAT3. Antioxidants & Redox Signaling, 22, 63–77. 10.1089/ars.2013.5748 25074339 PMC4270137

[acel12758-bib-0033] Pathan, M. , Keerthikumar, S. , Ang, C. S. , Gangoda, L. , Quek, C. Y. , Williamson, N. A. , … Mathivanan, S. (2015). FunRich: An open access standalone functional enrichment and interaction network analysis tool. Proteomics, 15, 2597–2601. 10.1002/pmic.201400515 25921073

[acel12758-bib-0034] Saltman, L. H. , Javed, A. , Ribadeneyra, J. , Hussain, S. , Young, D. W. , Osdoby, P. , … Bar‐Shavit, Z. (2005). Organization of transcriptional regulatory machinery in osteoclast nuclei: Compartmentalization of Runx1. Journal of Cellular Physiology, 204, 871–880. 10.1002/(ISSN)1097-4652 15828028

[acel12758-bib-0035] Sims, N. A. , & Walsh, N. C. (2012). Intercellular cross‐talk among bone cells: New factors and pathways. Current Osteoporosis Reports, 10, 109–117. 10.1007/s11914-012-0096-1 22427140

[acel12758-bib-0036] Trainor, P. A. , & Merrill, A. E. (2014). Ribosome biogenesis in skeletal development and the pathogenesis of skeletal disorders. Biochimica et Biophysica Acta, 1842, 769–778. 10.1016/j.bbadis.2013.11.010 24252615 PMC4020712

[acel12758-bib-0037] Vizcaino, J. A. , Csordas, A. , Del‐Toro, N. , Dianes, J. A. , Griss, J. , Lavidas, I. , … Hermjakob, H. (2016). 2016 update of the PRIDE database and its related tools. Nucleic Acids Research, 44, D447–D456. 10.1093/nar/gkv1145 26527722 PMC4702828

[acel12758-bib-0038] Xie, Y. , Chen, Y. , Zhang, L. , Ge, W. , Tang, P. (2017). The roles of bone‐derived exosomes and exosomal microRNAs in regulating bone remodelling. Journal of Cellular and Molecular Medicine 21, 1033–1041. 10.1111/jcmm.13039 27878944 PMC5387131

[acel12758-bib-0039] Yanez‐Mo, M. , Siljander, P. R. , Andreu, Z. , Zavec, A. B. , Borras, F. E. , Buzas, E. I. , … De Wever, O. (2015) Biological properties of extracellular vesicles and their physiological functions. Journal of Extracellular Vesicles 4, 27066 10.3402/jev.v4.27066 25979354 PMC4433489

[acel12758-bib-0040] Zu, Y. , Liang, X. , Du, J. , Zhou, S. , & Yang, C. (2015). Binding of integrin alpha1 to bone morphogenetic protein receptor IA suggests a novel role of integrin alpha1beta1 in bone morphogenetic protein 2 signalling. Journal of Biomechanics, 48, 3950–3954. 10.1016/j.jbiomech.2015.09.027 26475222

