# Supplementary material for: Involvement of serum‐derived exosomes of elderly patients with bone loss in failure of bone remodeling via alteration of exosomal bone‐related proteins
Source: Aging Cell. 2018 Mar 30;17(3):e12758. doi: 10.1111/acel.12758 (PMC5946082; doi:10.1111/acel.12758)
Supplement: Supplementary file 1 [file ACEL-17-e12758-s001.docx]

**
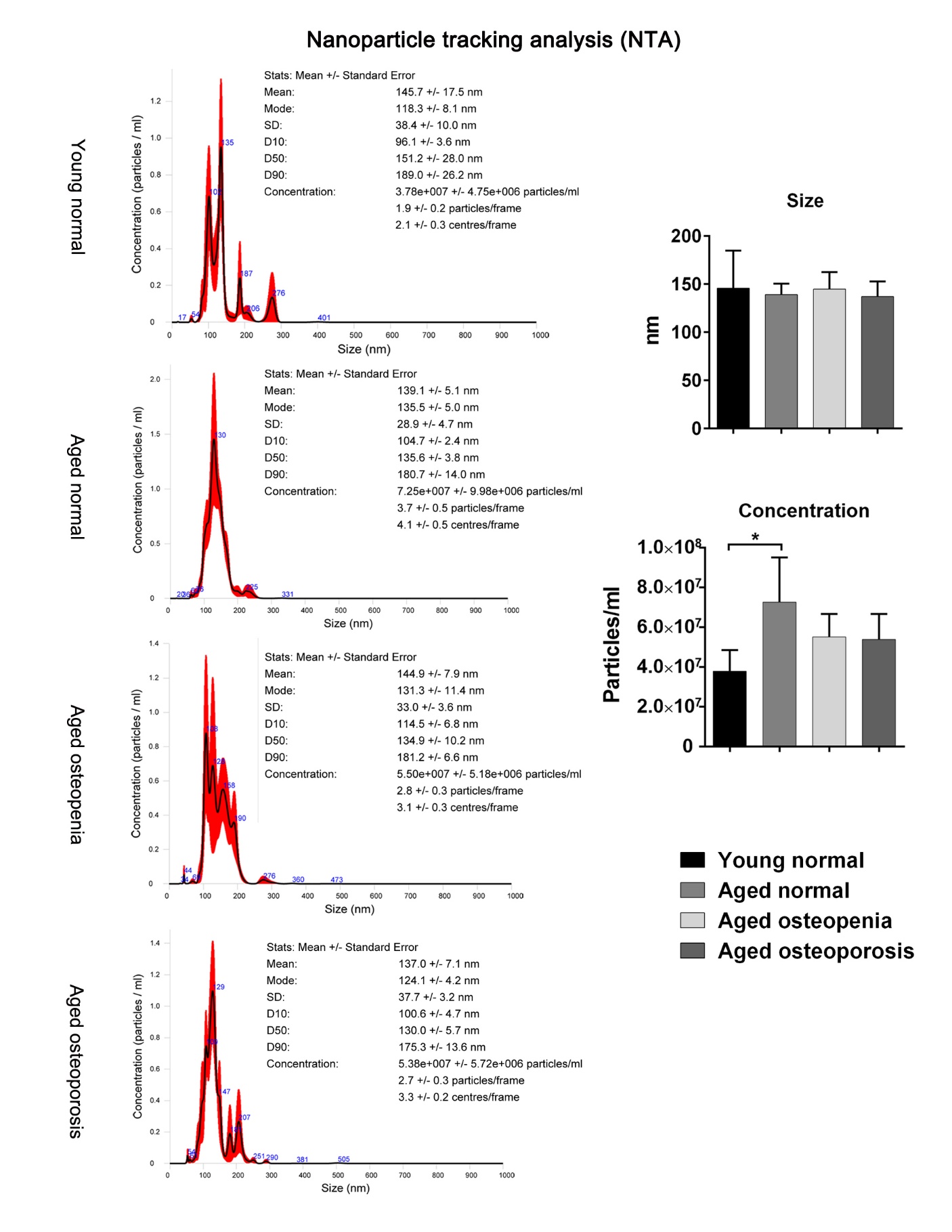
**

**Fig. S1.** **Nanoparticle Tracking Analysis.** Representative photographs are shown in the left panel. The size and concentration of SDEs are shown in the right panel. All values are representative of at least two independent experiments with similar results, and are displayed as mean ± SD. **P < 0.01, *P < 0.05.

**
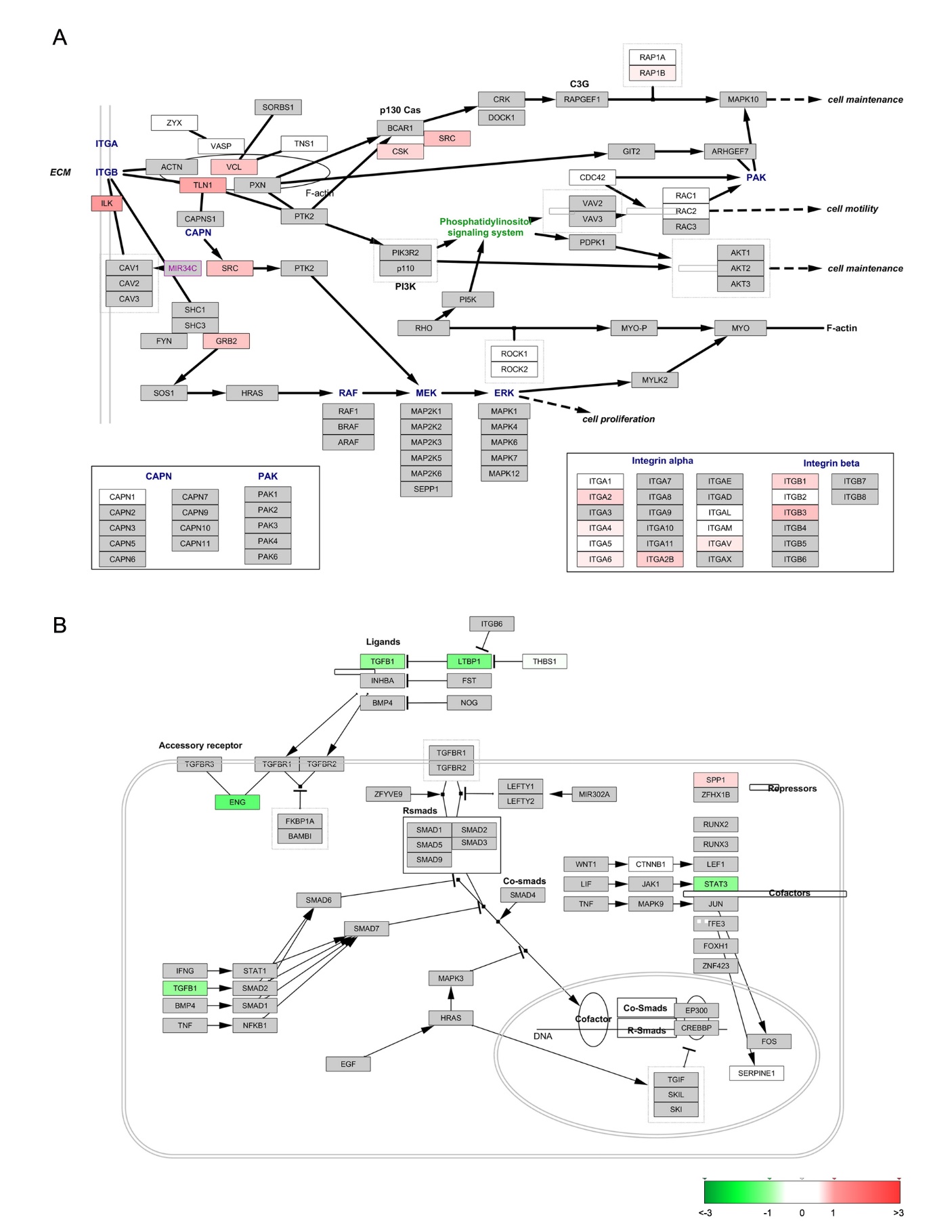
**

**Fig. S2. The DEPs of SDEs involved in Integrin-mediated cell adhesion pathways and** **TGF-β receptor signaling.** All identified proteins were mapped to the relevant Wiki pathway database with the ratio of 130/127 or 131/127. Proteins are represented by boxes labeled with the protein name. Relative protein expression levels in SDEs are indicated by colors. Proteins in gray were not identified in this study. (A) Integrin-mediated cell adhesion pathways of osteopenia patients; (B) TGF-β receptor signaling of osteoporosis patients.

**
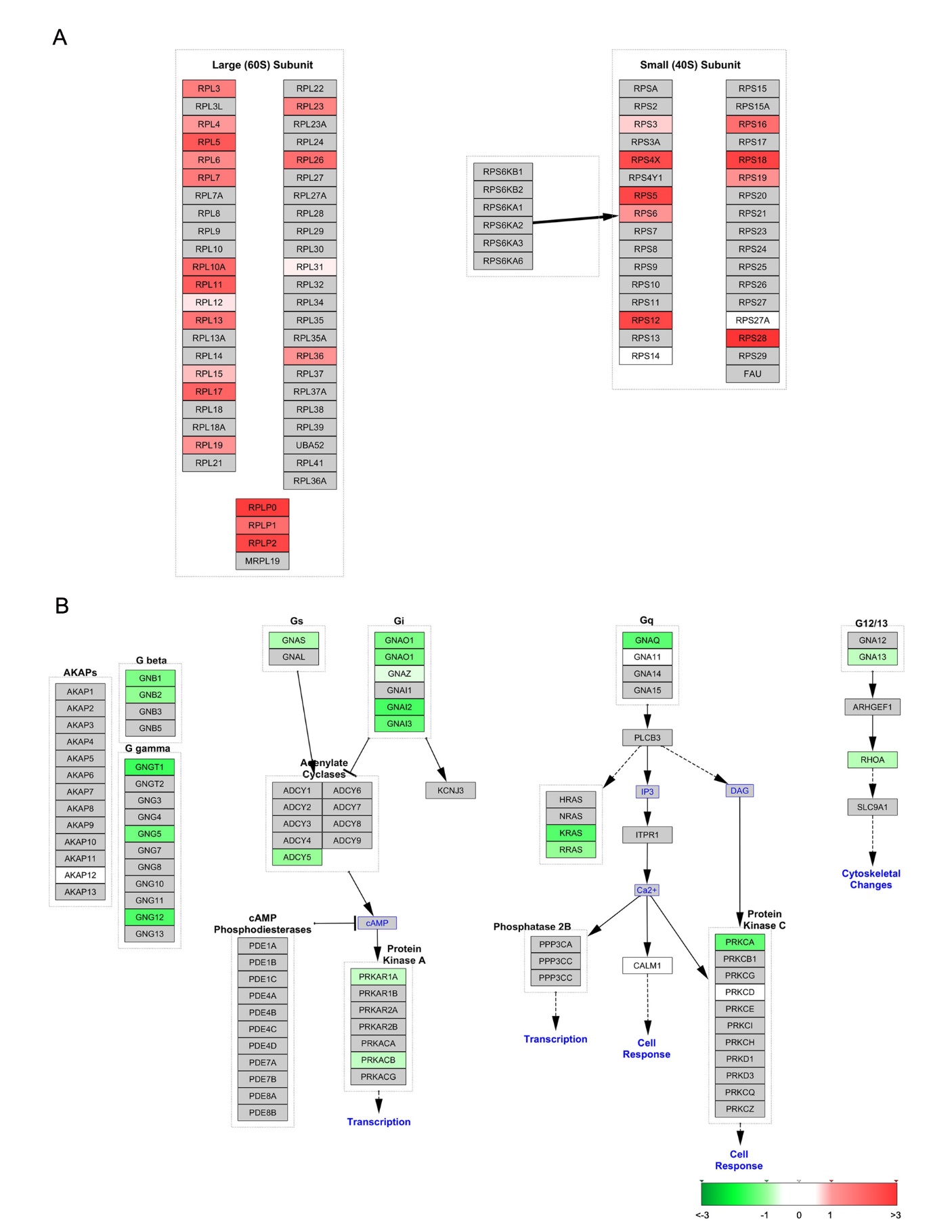
**

**Fig. S3. The DEPs of SDEs from osteopenia patients involved in the cytoplasmic ribosomal protein and G protein signaling pathways.** All identified proteins were mapped to the relevant Wiki pathway database with the ratio of 130/127 and 131/127. Proteins are represented by boxes labeled with the protein name. Relative protein expression levels in SDEs of osteoporosis patients are indicated by colors. Proteins in gray were not identified in this study. (A) Cytoplasmic ribosomal protein pathways; (B) G protein signaling pathways.

**
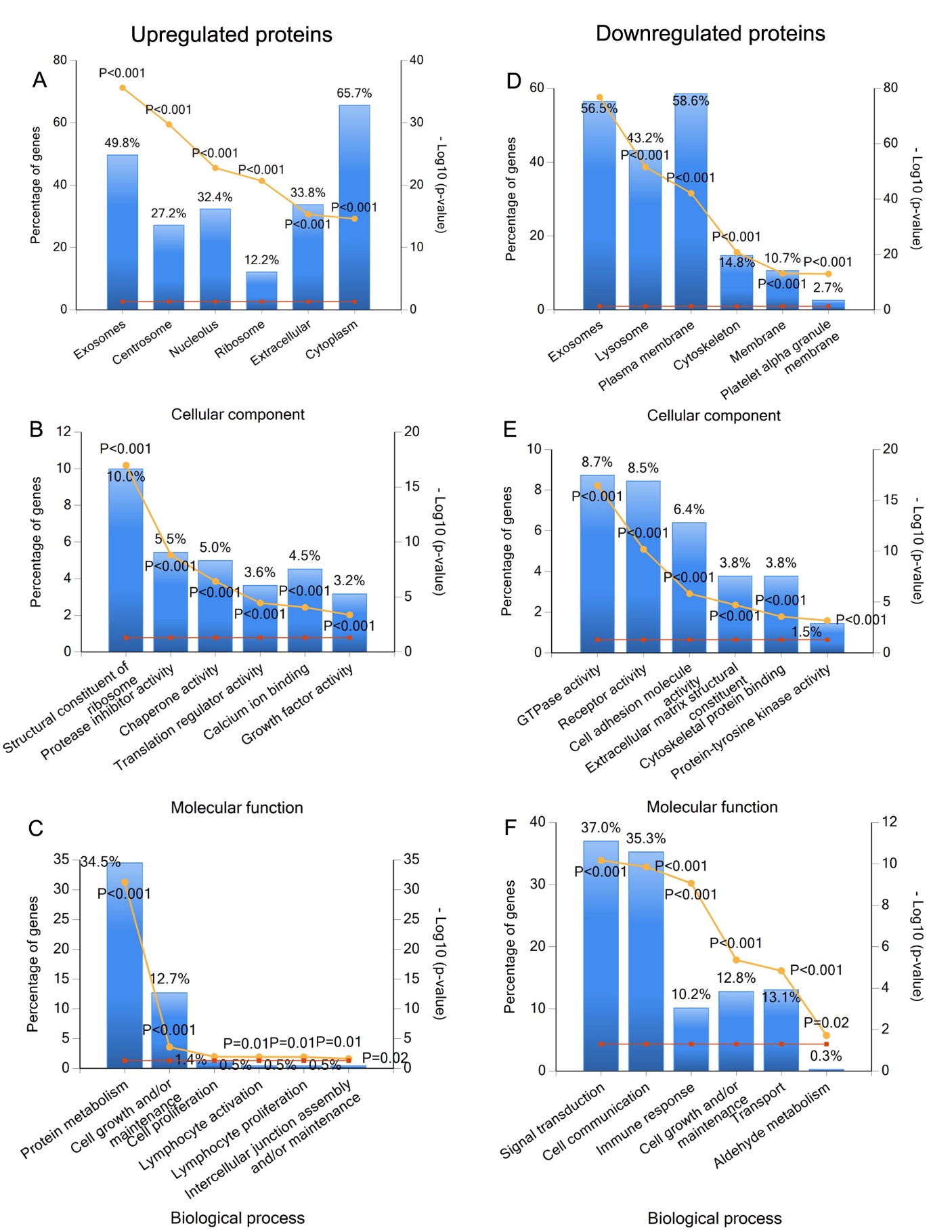
**

**Fig. S4. GO classification of DEPs from SDEs of patients with osteoporosis.** DEPs were submitted to the GO classification system. The six most enriched categories and the enrichment significance (−log (P-value), P < 0.05) in cellular components, molecular functions and biological process categories are shown. The percentage of proteins identified in each category is indicated. (A-C) Analysis of upregulated proteins in cellular components, molecular functions and biological process categories, respectively. (D-F) Analysis of downregulated proteins in cellular components, molecular functions and biological process categories, respectively.


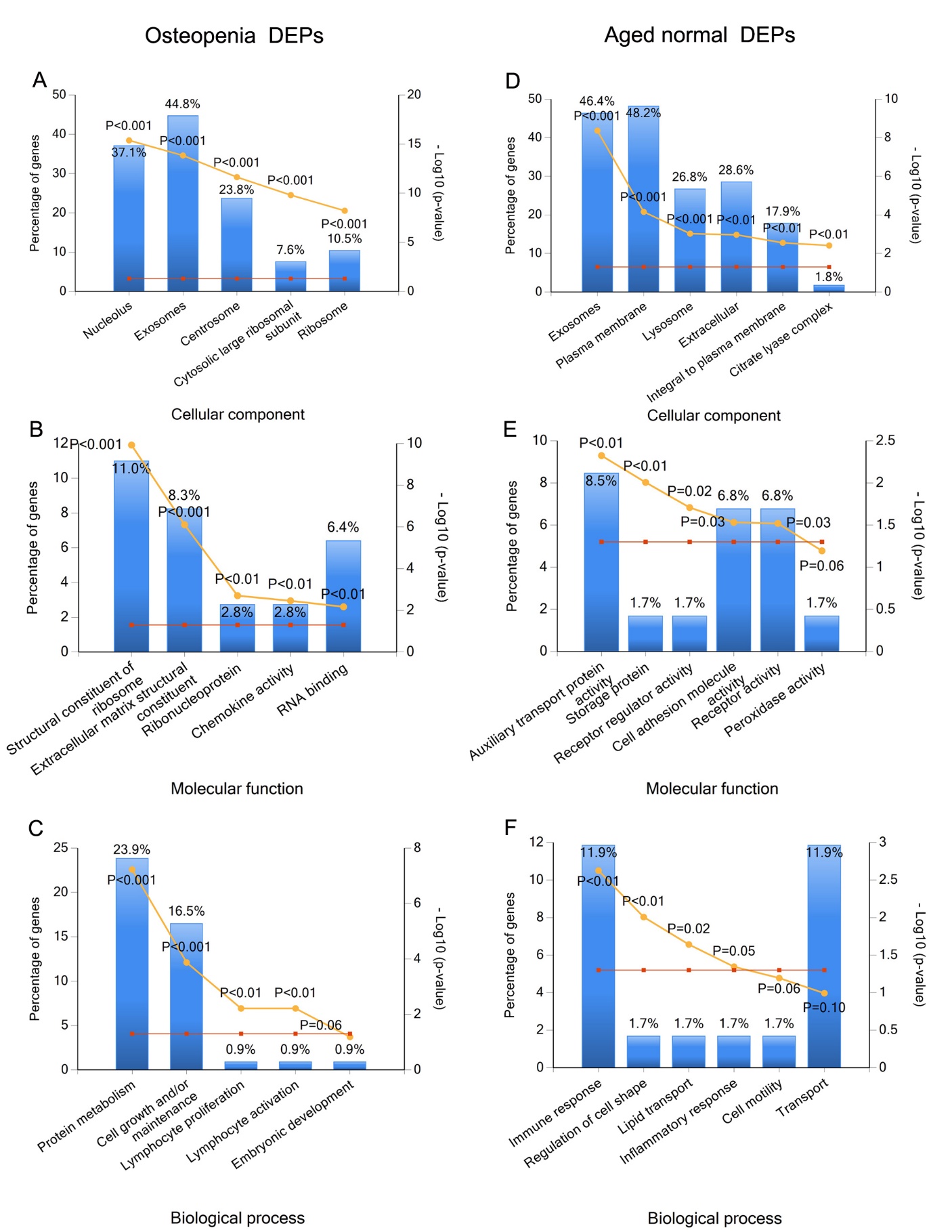


**Fig. S5.** **GO classification of DEPs from SDEs of patients with osteopenia and aged volunteers.** DEPs were submitted to the GO classification system. The six most enriched categories in cellular components, molecular functions and biological process and the enrichment significance (−log (*P*-value), *P* < 0.05) are shown. The percentage of proteins identified in each category is indicated. (A–C) Analysis of DEPs from SDEs of osteopenia patients in cellular components, molecular functions and biological process, respectively. (D–F) Analysis of DEPs from SDEs of aged volunteers in cellular components, molecular functions and biological process, respectively.

**
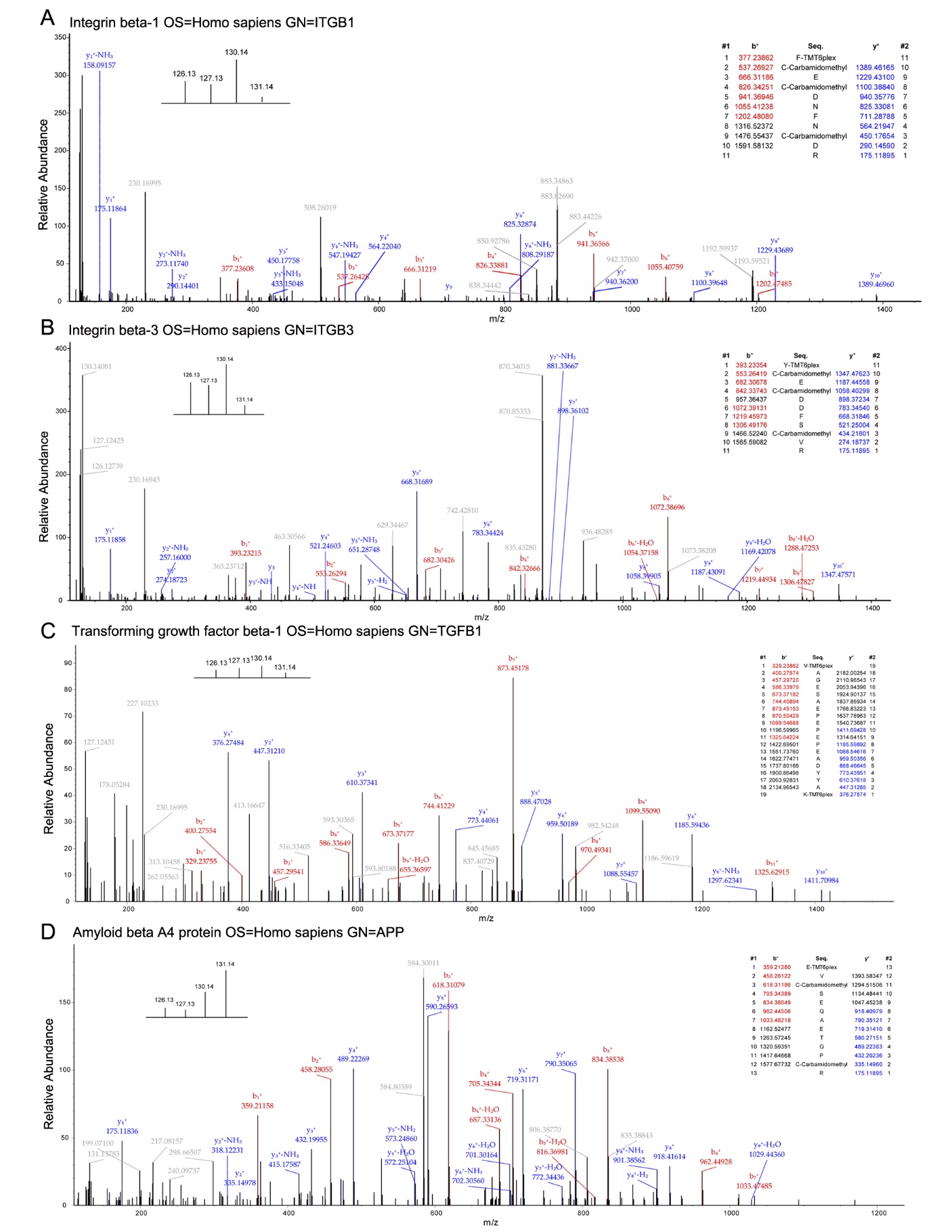
**

**Fig. S6.** **Validation of the expression level of DEPs by representative MS/MS spectral data.** (A–D) Representative mass spectral data of identified proteins: integrin β_1_, integrin β_3_, TGFβ_1_ and APP.


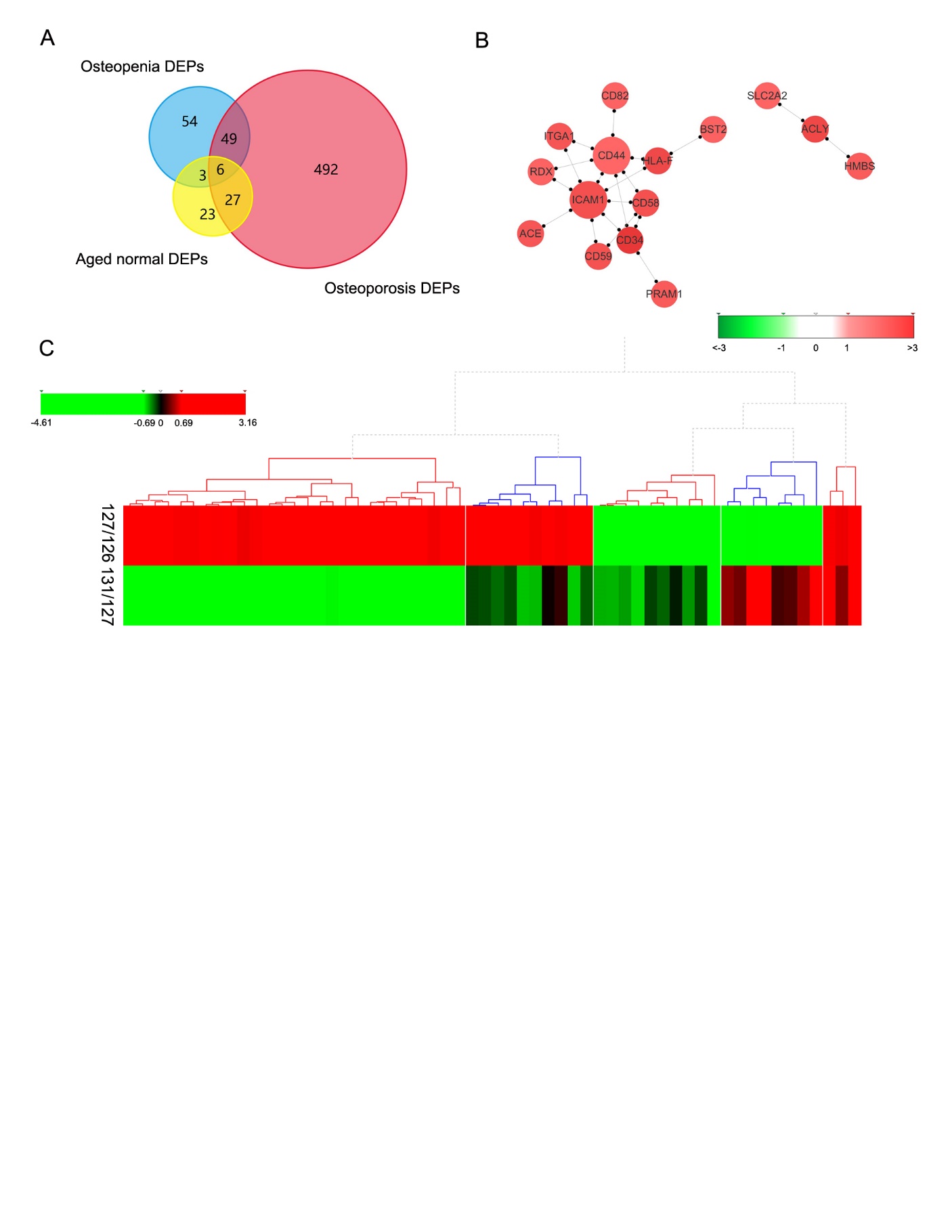


**Fig. S7. Analysis of the association between aged normal DEPs and osteoporosis DEPs.** (A) Venn diagram showing the overlap of aged normal DEPs with osteoporosis or osteopenia DEPs. Numbers represent the number of Entrez Gene IDs. (B) Network of overlapping DEPs in SDEs of patients with osteoporosis and aged normal volunteers. (C) Heatmap of DEPs of SDEs from patients with osteoporosis (131/127) and aged normal volunteers (127/126).
