# Supplementary material for: Involvement of serum‐derived exosomes of elderly patients with bone loss in failure of bone remodeling via alteration of exosomal bone‐related proteins
Source: Aging Cell. 2018 Mar 30;17(3):e12758. doi: 10.1111/acel.12758 (PMC5946082; doi:10.1111/acel.12758)
Supplement: Supplementary file 6 [file ACEL-17-e12758-s006.docx]

**Supplementary Table 5A. Biological pathways involving DEPs from SDEs of patients with osteoporosis**

| **Pathways** | **Upregulated proteins** | | |
| --- | --- | --- | --- |
|  | **Gene Count** | **Gene Symbol** | **FDR** |
| Cytoplasmic Ribosomal Proteins | 24 | RPL36 RPL10A RPL3 RPL4 RPL5 RPL6 RPL7 RPL11 RPL13 RPL17 RPL19 RPL26 RPLP0 RPLP1 RPLP2 RPS4X RPS5 RPS6 RPS12 RPS16 RPS18 RPS19 RPS28 RPL23 | 0e+00 |
| Blood Clotting Cascade | 5 | F2 F7 F9 F10 SERPINB2 | 2.12e-02 |
| Translation Factors | 7 | EEF1D EEF2 EIF2S3 EIF4G1 EIF5A EIF3D EIF2S2 | 2.12e-02 |
| Complement and Coagulation Cascades | 7 | F2 F7 F9 F10 PROC PROS1 TFPI | 2.54e-02 |
| Cori Cycle | 3 | LDHA PGAM1 TPI1 | 3.19e-01 |
| Photodynamic therapy-induced unfolded protein response | 5 | HSPA5 NARS DNAJC3 HSP90B1 CALR | 2.54e-02 |
| Aryl Hydrocarbon Receptor Pathway | 4 | PTGES3 HSP90AA1 IGFBP1 SERPINB2 | 1e+00 |
| Parkin-Ubiquitin Proteasomal System pathway | 5 | HSPA2 HSPA5 PSMC3 PSMD2 PSMD6 | 1e+00 |
| Glycolysis and Gluconeogenesis | 4 | ALDOB LDHA PGAM1 TPI1 | 1e+00 |
| Selenium Micronutrient Network | 5 | F2 F7 PRDX1 SOD3 TXN | 1e+00 |

P< 0.05 was considered statistically significant. False discovery rate (FDR).

**Supplementary Table 5B. Biological pathways involving DEPs from SDEs of patients with osteoporosis**

| **Pathways** | **Downregulated proteins** | | |
| --- | --- | --- | --- |
|  | **Gene Count** | **Gene Symbol** | **FDR** |
| Integrin-mediated Cell Adhesion | 20 | CSK GRB2 ILK ITGA6 ITGA1 ITGA2 ITGA2B ITGA4 ITGAM ITGB1 ITGB2 ITGB3 RAC1 RAC2 RAP1A RAP1B SRC TLN1 TNS1 CDC42 | 3.92e-08 |
| Focal Adhesion | 25 | COL6A2 FLNA FN1 GRB2 PARVB TNC ILK ITGA6 ITGA2  ITGA2B ITGA4 ITGAM ITGB1 ITGB2 ITGB3 RELN RAC1 RAC2 RAP1A RAP1B ACTB SRC TLN1 VWF CDC42 | 8.51e-07 |
| G Protein Signaling Pathways | 13 | ADCY5 GNAI2 GNAI3 GNAO1 GNAQ GNB1 GNB2 GNG5  GNGT1 KRAS PRKCA GNG12 RRAS | 1.65e-03 |
| RalA downstream regulated genes | 5 | KRAS RAC1 RAC2 RALA CDC42 | 1.97e-03 |
| Nanoparticle-mediated activation of receptor signaling | 7 | FN1 GRB2 ITGA1 ITGB1 KRAS SRC TLN1 | 1.97e-03 |
| EGF/EGFR Signaling Pathway | 17 | CFL1 CSK EPS8 GRB2 KRAS STMN1 PLSCR1 PRKCA  RAC1 RALA RALB RAP1A SRC STAT3 DOK2 USP8 CDC42 | 1.97e-03 |
| Calcium Regulation in the Cardiac Cell | 16 | ADCY5 GNAI2 GNAI3 GNAO1 GNAQ GNB1 GNB2 GNG5  GNGT1 ANXA6 ARRB1 ATP2A3 GNG2 PRKCA GNG12 GNB4 | 2.18e-03 |
| Human Thyroid Stimulating Hormone (TSH) signaling pathway | 10 | GNAI2 GNAI3 GNAO1 GNAQ GNB1 GNG2 RAP1A RAP1B SRC STAT3 | 3.07e-03 |
| Rac1/Pak1/p38/MMP-2 pathway | 10 | FN1 ANGPT1 GRB2 ITGB1 KRAS STMN1 RAC1 SRC STAT3 DOK2 | 3.12e-03 |
| Signaling of Hepatocyte Growth Factor Receptor | 7 | GRB2 ITGA1 ITGB1 RAP1A RAP1B SRC STAT3 | 4.23e-03 |

P< 0.05 was considered statistically significant. False discovery rate (FDR).

Red represents DEPs derived from plasma membrane.

**Supplementary Table 5C. Biological pathways involving DEPs from SDEs of patients with osteopenia**

| **Pathways** | **DEPs** | | |
| --- | --- | --- | --- |
|  | **Gene Count** | **Gene Symbol** | **FDR** |
| Cytoplasmic Ribosomal Proteins | 13 | RPL36 RPL10A RPL3 RPL4 RPL6 RPL7 RPL15 RPL17 RPL19 RPLP0 RPLP2 RPS5 RPS6 | 3.69e-09 |
| mRNA Processing | 8 | HNRNPH1 HNRNPK SRSF1 SRSF2 SRSF4 SRSF6 SRSF7 TRA2B | 1.37e-02 |

P< 0.05 was considered statistically significant. False discovery rate (FDR).

**Supplementary Table 5D. Biological pathways involving DEPs from SDEs of elderly volunteers**

| **Pathways** | **DEPs** | | |
| --- | --- | --- | --- |
|  | **Gene Count** | **Gene Symbol** | **FDR** |
| Selenium Micronutrient Network | 3 | ICAM1 SAA1 PRDX2 | 1e+00 |

P< 0.05 was considered statistically significant. False discovery rate (FDR).
