# Supplementary material for: Involvement of serum‐derived exosomes of elderly patients with bone loss in failure of bone remodeling via alteration of exosomal bone‐related proteins
Source: Aging Cell. 2018 Mar 30;17(3):e12758. doi: 10.1111/acel.12758 (PMC5946082; doi:10.1111/acel.12758)
Supplement: Supplementary file 7 [file ACEL-17-e12758-s007.docx]

**Supplementary Table 6A. Information about volunteers recruited for experiments**

| TMT Label | Gender | Age | Diagnosis | BMD  (g/cm^2^) | Z value | Collection date |
| --- | --- | --- | --- | --- | --- | --- |
| **126**  **(Young**  **Normal)** | female | 49 | Within the expected range for age | 0.367 | -1.26 | 2015.09.25 |
|  | female | 46 | Within the expected range for age | 0.524 | 1.21 | 2015.09.25 |
|  | female | 44 | Within the expected range for age | 0.428 | -0.43 | 2015.09.24 |
|  | female | 44 | Within the expected range for age | 0.383 | -1.18 | 2015.10.16 |
|  | male | 44 | Within the expected range for age | 0.558 | -0.04 | 2015.10.23 |
|  | male | 43 | Within the expected range for age | 0.48 | -1.36 | 2015.10.23 |
|  | female | 42 | Within the expected range for age | 0.501 | 0.67 | 2015.09.25 |
|  | female | 42 | Within the expected range for age | 0.425 | -0.57 | 2015.09.25 |
|  | male | 39 | Within the expected range for age | 0.647 | 1.25 | 2015.10.23 |
|  | female | 38 | Within the expected range for age | 0.468 | -1.58 | 2015.09.25 |
|  | female | 35 | Within the expected range for age | 0.452 | -0.4 | 2015.09.24 |
|  | male | 35 | Within the expected range for age | 0.576 | -0.09 | 2015.10.23 |
|  | male | 32 | Within the expected range for age | 0.55 | -0.45 | 2015.10.17 |
|  | female | 31 | Within the expected range for age | 0.372 | -0.45 | 2015.09.25 |
|  | male | 31 | Within the expected range for age | 0.565 | -0.28 | 2015.10.17 |
|  | male | 31 | Within the expected range for age | 0.522 | -0.99 | 2015.10.23 |
|  | male | 30 | Within the expected range for age | 0.473 | -1.81 | 2015.10.23 |
|  | male | 29 | Within the expected range for age | 0.463 | -1.52 | 2015.10.23 |
|  | male | 29 | Within the expected range for age | 0.482 | -1.67 | 2015.10.23 |
|  | female | 28 | Within the expected range for age | 0.431 | -0.77 | 2015.09.25 |
|  | male | 28 | Within the expected range for age | 0.553 | -0.47 | 2015.10.17 |
|  | male | 28 | Within the expected range for age | 0.543 | -0.65 | 2015.10.23 |
|  | male | 27 | Within the expected range for age | 0.478 | -1.73 | 2015.10.23 |
|  | female | 26 | Within the expected range for age | 0.452 | -0.42 | 2015.09.24 |
|  | female | 26 | Within the expected range for age | 0.411 | -1.11 | 2015.09.25 |
|  | female | 26 | Within the expected range for age | 0.441 | -0.6 | 2015.09.25 |
|  | male | 26 | Within the expected range for age | 0.503 | -1.31 | 2015.10.17 |
|  | male | 26 | Within the expected range for age | 0.584 | 0.04 | 2015.10.23 |
|  | male | 26 | Within the expected range for age | 0.561 | -0.34 | 2015.10.23 |
|  | male | 26 | Within the expected range for age | 0.521 | -1.01 | 2015.10.23 |
|  | male | 26 | Within the expected range for age | 0.526 | -0.93 | 2015.10.23 |
|  | male | 25 | Within the expected range for age | 0.473 | -1.8 | 2015.10.17 |
|  | male | 25 | Within the expected range for age | 0.528 | -0.9 | 2015.10.23 |
|  | male | 25 | Within the expected range for age | 0.527 | -0.91 | 2015.10.23 |
|  | female | 23 | Within the expected range for age | 0.413 | -1.06 | 2015.09.25 |
|  | female | 21 | Within the expected range for age | 0.387 | -1.49 | 2015.09.23 |
|  |  |  |  |  |  |  |

**Supplementary Table 6B. Information about volunteers recruited for experiments**

| TMT Label | Gender | Age | Diagnosis | BMD  (g/cm^2^) | T value | Collection date |
| --- | --- | --- | --- | --- | --- | --- |
| **127**  **(Aged**  **Normal)** | female | 57 | Normal | 0.521 | 0.73 | 2015.10.30 |
|  | female | 61 | Normal | 0.459 | -0.29 | 2015.10.30 |
|  | female | 63 | Normal | 0.456 | -0.35 | 2015.11.2 |
|  | female | 56 | Normal | 0.541 | 1.06 | 2015.11.2 |
|  | female | 60 | Normal | 0.454 | -0.39 | 2015.11.4 |
|  | female | 58 | Normal | 0.457 | -0.33 | 2015.11.11 |
|  | female | 57 | Normal | 0.428 | -0.98 | 2015.11.11 |
|  | female | 56 | Normal | 0.457 | -0.34 | 2015.11.13 |
|  | female | 57 | Normal | 0.468 | -0.16 | 2015.11.16 |
|  | male | 60 | Normal | 0.569 | -0.21 | 2015.10.29 |
|  | male | 68 | Normal | 0.609 | 0.46 | 2015.11.2 |
|  | male | 66 | Normal | 0.565 | -0.28 | 2015.11.20 |
|  | male | 65 | Normal | 0.558 | -0.39 | 2015.11.24 |
|  | male | 60 | Normal | 0.600 | 0.30 | 2015.11.24 |
|  | male | 61 | Normal | 0.544 | -0.63 | 2015.11.27 |
|  | male | 60 | Normal | 0.597 | 0.26 | 2015.11.27 |
|  | male | 63 | Normal | 0.552 | -0.49 | 2015.12.1 |
|  | male | 62 | Normal | 0.538 | -0.72 | 2015.12.2 |
|  | male | 61 | Normal | 0.573 | -0.14 | 2015.12.4 |
|  | male | 60 | Normal | 0.550 | -0.53 | 2015.12.4 |
|  | male | 61 | Normal | 0.616 | 1.66 | 2015.12.7 |
|  | male | 70 | Normal | 0.522 | -0.99 | 2015.12.8 |
|  | male | 69 | Normal | 0.653 | 1.19 | 2015.12.9 |
|  | male | 63 | Normal | 0.550 | -0.52 | 2015.12.9 |
|  | male | 61 | Normal | 0.588 | 0.12 | 2015.12.11 |
|  | male | 62 | Normal | 0.736 | 2.58 | 2015.12.14 |
|  |  |  |  |  |  |  |

**Supplementary Table 6C. Information about patients recruited for experiments**

| TMT Label | Gender | Age | Diagnosis | BMD  (g/cm^2^) | T value | | Collection date |
| --- | --- | --- | --- | --- | --- | --- | --- |
| **130**  **(Aged**  **Osteopenia)** | male | 84 | Osteopenia | 0.434 | | -2.45 | 2015.10.23 |
|  | male | 74 | Osteopenia | 0.476 | | -1.75 | 2015.10.19 |
|  | male | 73 | Osteopenia | 0.375 | | -1.71 | 2015.10.19 |
|  | male | 69 | Osteopenia | 0.492 | | -1.49 | 2015.11.2 |
|  | female | 68 | Osteopenia | 0.339 | | -2.30 | 2015.10.22 |
|  | female | 68 | Osteopenia | 0.338 | | -2.31 | 2015.10.20 |
|  | male | 68 | Osteopenia | 0.502 | | -1.33 | 2015.12.2 |
|  | male | 67 | Osteopenia | 0.521 | | -1.01 | 2015.12.9 |
|  | female | 66 | Osteopenia | 0.415 | | -1.03 | 2015.10.20 |
|  | female | 66 | Osteopenia | 0.414 | | -1.04 | 2015.11.9 |
|  | male | 66 | Osteopenia | 0.490 | | -1.52 | 2015.12.4 |
|  | female | 65 | Osteopenia | 0.340 | | -2.29 | 2015.10.30 |
|  | male | 65 | Osteopenia | 0.444 | | -2.29 | 2015.11.5 |
|  | male | 65 | Osteopenia | 0.480 | | -1.68 | 2015.11.5 |
|  | female | 64 | Osteopenia | 0.347 | | -2.17 | 2015.11.2 |
|  | male | 64 | Osteopenia | 0.483 | | -1.64 | 2015.12.9 |
|  | male | 64 | Osteopenia | 0.481 | | -1.67 | 2015.12.14 |
|  | male | 64 | Osteopenia | 0.472 | | -1.82 | 2015.12.18 |
|  | female | 63 | Osteopenia | 0.353 | | -2.06 | 2015.11.9 |
|  | male | 63 | Osteopenia | 0.490 | | -1.52 | 2015.11.17 |
|  | female | 62 | Osteopenia | 0.383 | | -1.56 | 2015.11.9 |
|  | female | 62 | Osteopenia | 0.320 | | -2.08 | 2015.11.16 |
|  | male | 62 | Osteopenia | 0.463 | | -1.98 | 2015.11.4 |
|  | male | 62 | Osteopenia | 0.456 | | -2.10 | 2015.11.20 |
|  | male | 62 | Osteopenia | 0.490 | | -1.52 | 2015.12.3 |
|  | male | 62 | Osteopenia | 0.480 | | -1.69 | 2015.12.8 |
|  | male | 62 | Osteopenia | 0.467 | | -1.91 | 2015.12.9 |
|  | male | 62 | Osteopenia | 0.502 | | -1.33 | 2015.12.14 |
|  | female | 61 | Osteopenia | 0.338 | | -2.32 | 2015.11.4 |
|  | male | 61 | Osteopenia | 0.511 | | -1.17 | 2015.11.2 |
|  | male | 61 | Osteopenia | 0.468 | | -1.88 | 2015.12.8 |
|  | male | 61 | Osteopenia | 0.476 | | -1.75 | 2015.12.15 |
|  | female | 60 | Osteopenia | 0.398 | | -1.32 | 2015.10.29 |
|  | female | 60 | Osteopenia | 0.378 | | -1.65 | 2015.10.29 |
|  | male | 60 | Osteopenia | 0.488 | | -1.57 | 2015.10.29 |
|  | male | 60 | Osteopenia | 0.449 | | -2.21 | 2015.10.30 |
|  | male | 60 | Osteopenia | 0.486 | | -1.60 | 2015.12.10 |
|  | female | 59 | Osteopenia | 0.360 | | -1.96 | 2015.11.4 |
|  | female | 59 | Osteopenia | 0.376 | | -1.68 | 2015.11.16 |
|  | female | 58 | Osteopenia | 0.412 | | -1.09 | 2015.10.29 |
|  | female | 57 | Osteopenia | 0.370 | | -1.78 | 2015.11.4 |
|  | female | 56 | Osteopenia | 0.362 | | -1.92 | 2015.11.10 |
|  | female | 56 | Osteopenia | 0.367 | | -1.84 | 2015.11.11 |
|  | female | 56 | Osteopenia | 0.348 | | -2.14 | 2015.11.13 |
|  | female | 55 | Osteopenia | 0.397 | | -1.33 | 2015.11.4 |
|  | female | 55 | Osteopenia | 0.369 | | -1.80 | 2015.11.10 |
|  |  |  |  |  | |  |  |

**Supplementary Table 6D. Information about patients recruited for experiments**

| TMT Label | Gender | Age | Diagnosis | BMD  (g/cm^2^) | T value | Collection date |
| --- | --- | --- | --- | --- | --- | --- |
| **131**  **(Aged**  **Osteoporosis)** | female | 70 | Osteoporosis | 0.167 | -5.17 | 2015.11.9 |
|  | female | 69 | Osteoporosis | 0.220 | -4.28 | 2015.10.23 |
|  | female | 69 | Osteoporosis | 0.203 | -4.56 | 2015.10.22 |
|  | female | 69 | Osteoporosis | 0.165 | -5.20 | 2015.10.29 |
|  | female | 69 | Osteoporosis | 0.173 | -5.07 | 2015.11.2 |
|  | female | 68 | Osteoporosis | 0.253 | -3.74 | 2015.10.20 |
|  | female | 68 | Osteoporosis | 0.228 | -4.15 | 2015.11.13 |
|  | female | 67 | Osteoporosis | 0.314 | -2.71 | 2015.10.22 |
|  | female | 67 | Osteoporosis | 0.255 | -3.69 | 2015.10.21 |
|  | female | 67 | Osteoporosis | 0.280 | -3.28 | 2015.10.21 |
|  | male | 67 | Osteoporosis | 0.426 | -2.60 | 2015.11.20 |
|  | male | 66 | Osteoporosis | 0.339 | -0.45 | 2015.12.1 |
|  | female | 65 | Osteoporosis | 0.290 | -3.11 | 2015.10.22 |
|  | female | 65 | Osteoporosis | 0.283 | -3.23 | 2015.10.28 |
|  | female | 64 | Osteoporosis | 0.303 | -2.89 | 2015.10.28 |
|  | female | 64 | Osteoporosis | 0.073 | -6.73 | 2015.11.12 |
|  | female | 63 | Osteoporosis | 0.267 | -3.51 | 2015.10.28 |
|  | female | 62 | Osteoporosis | 0.198 | -4.64 | 2015.10.28 |
|  | male | 62 | Osteoporosis | 0.369 | -3.53 | 2015.12.14 |
|  | male | 62 | Osteoporosis | 0.426 | -2.59 | 2015.12.15 |
|  | female | 61 | Osteoporosis | 0.254 | -3.71 | 2015.10.28 |
|  | female | 61 | Osteoporosis | 0.279 | -3.30 | 2015.11.2 |
|  | male | 61 | Osteoporosis | 0.397 | -3.08 | 2015.12.1 |
|  | female | 60 | Osteoporosis | 0.255 | -3.71 | 2015.10.28 |
|  | male | 60 | Osteoporosis | 0.428 | -2.56 | 2015.12.1 |
|  | male | 60 | Osteoporosis | 0.386 | -3.26 | 2015.12.14 |
|  | male | 60 | Osteoporosis | 0.382 | -3.32 | 2015.12.15 |
|  | female | 59 | Osteoporosis | 0.280 | -3.28 | 2015.11.2 |
|  | female | 58 | Osteoporosis | 0.279 | -3.30 | 2015.11.2 |
|  | female | 57 | Osteoporosis | 0.189 | -4.80 | 2015.10.30 |
|  | female | 57 | Osteoporosis | 0.258 | -3.65 | 2015.11.10 |
|  |  |  |  |  |  |  |
