# Supplementary material for: Involvement of serum‐derived exosomes of elderly patients with bone loss in failure of bone remodeling via alteration of exosomal bone‐related proteins
Source: Aging Cell. 2018 Mar 30;17(3):e12758. doi: 10.1111/acel.12758 (PMC5946082; doi:10.1111/acel.12758)
Supplement: Supplementary file 8 [file ACEL-17-e12758-s008.docx]

**Supplementary Table 7A. Information about volunteers recruited for experiments**

| Young  Normal | Gender | Age | Diagnosis | BMD  (g/cm^2^) | Z value | Collection date |
| --- | --- | --- | --- | --- | --- | --- |
|  | female | 29 | Within the expected range for age | 0.441 | -0.6 | 2017.10.23 |
|  | female | 42 | Within the expected range for age | 0.468 | -1.58 | 2017.09.25 |
|  | female | 34 | Within the expected range for age | 0.413 | -1.06 | 2017.09.25 |
|  | female | 35 | Within the expected range for age | 0.375 | -1.71 | 2017.10.23 |
|  | female | 44 | Within the expected range for age | 0.401 | -1.26 | 2017.09.25 |
|  | female | 43 | Within the expected range for age | 0.461 | -0.07 | 2017.10.23 |
|  | male | 41 | Within the expected range for age | 0.528 | -0.9 | 2017.09.25 |
|  | male | 42 | Within the expected range for age | 0.508 | -1.22 | 2017.09.25 |
|  | male | 39 | Within the expected range for age | 0.605 | 0.39 | 2017.10.23 |
|  | male | 39 | Within the expected range for age | 0.528 | -0.9 | 2017.09.24 |
|  | male | 35 | Within the expected range for age | 0.527 | -0.91 | 2017.10.23 |
|  | male | 32 | Within the expected range for age | 0.576 | -0.09 | 2017.10.17 |
|  | female | 31 | Within the expected range for age | 0.433 | -0.73 | 2017.09.25 |
|  | female | 33 | Within the expected range for age | 0.433 | -0.73 | 2017.10.17 |
|  | female | 31 | Within the expected range for age | 0.486 | 0.15 | 2017.10.23 |
|  | male | 30 | Within the expected range for age | 0.565 | -0.28 | 2017.10.23 |
|  | male | 29 | Within the expected range for age | 0.468 | -1.58 | 2017.10.23 |
|  | male | 39 | Within the expected range for age | 0.584 | 0.04 | 2017.10.23 |
|  |  |  |  |  |  |  |

**Supplementary Table 7B. Information about volunteers recruited for experiments**

| Aged  Normal | Gender | Age | Diagnosis | BMD  (g/cm^2^) | T value | Collection date |
| --- | --- | --- | --- | --- | --- | --- |
|  | female | 60 | Normal | 0.428 | -0.98 | 2017.10.29 |
|  | male | 58 | Normal | 0.544 | -0.63 | 2017.10.29 |
|  | male | 57 | Normal | 0.653 | 1.19 | 2017.11.13 |
|  | male | 56 | Normal | 0.55 | -0.52 | 2017.11.16 |
|  | male | 60 | Normal | 0.588 | 0.12 | 2017.11.13 |
|  | male | 58 | Normal | 0.617 | 0.59 | 2017.11.11 |
|  | male | 57 | Normal | 0.561 | -0.34 | 2017.11.11 |
|  | male | 56 | Normal | 0.554 | -0.46 | 2017.11.13 |
|  | female | 57 | Normal | 0.479 | 0.03 | 2017.11.11 |
|  | female | 56 | Normal | 0.458 | -0.32 | 2017.11.2 |
|  | female | 66 | Normal | 0.484 | 0.12 | 2017.11.20 |
|  | female | 65 | Normal | 0.436 | -0.69 | 2017.11.24 |
|  | female | 60 | Normal | 0.421 | -0.94 | 2017.11.13 |
|  | male | 61 | Normal | 0.547 | -0.57 | 2017.11.16 |
|  | male | 60 | Normal | 0.856 | 4.57 | 2017.10.29 |
|  | female | 67 | Normal | 0.513 | 0.61 | 2017.11.2 |
|  | female | 62 | Normal | 0.427 | -0.83 | 2017.11.13 |
|  |  |  |  |  |  |  |

**Supplementary Table 7C. Information about patients recruited for experiments**

| Aged  Osteopenia | Gender | Age | Diagnosis | BMD  (g/cm^2^) | T value | | Collection date |
| --- | --- | --- | --- | --- | --- | --- | --- |
|  | female | 67 | Osteopenia | 0.476 | | -1.96 | 2017.12.9 |
|  | female | 66 | Osteopenia | 0.49 | | -1.78 | 2017.12.12 |
|  | female | 66 | Osteopenia | 0.521 | | -2.14 | 2017.12.11 |
|  | male | 69 | Osteopenia | 0.502 | | -1.75 | 2017.11.9 |
|  | male | 77 | Osteopenia | 0.458 | | -1.52 | 2017.10.22 |
|  | male | 74 | Osteopenia | 0.487 | | -1.01 | 2017.10.20 |
|  | male | 68 | Osteopenia | 0.475 | | -1.33 | 2017.12.2 |
|  | male | 67 | Osteopenia | 0.466 | | -2.06 | 2017.12.9 |
|  | male | 66 | Osteopenia | 0.559 | | -1.58 | 2017.10.20 |
|  | female | 66 | Osteopenia | 0.492 | | -1.79 | 2017.11.9 |
|  | female | 64 | Osteopenia | 0.37 | | -2.04 | 2017.10.30 |
|  | female | 63 | Osteopenia | 0.36 | | -1.91 | 2017.11.5 |
|  | female | 64 | Osteopenia | 0.348 | | -2.01 | 2017.11.5 |
|  | male | 56 | Osteopenia | 0.37 | | -1.78 | 2017.12.2 |
|  | male | 64 | Osteopenia | 0.355 | | -1.93 | 2017.12.9 |
|  | male | 58 | Osteopenia | 0.369 | | -1.04 | 2017.10.20 |
|  | female | 64 | Osteopenia | 0.357 | | -1.15 | 2017.11.9 |
|  | female | 63 | Osteopenia | 0.408 | | -1.97 | 2017.11.9 |
|  | male | 63 | Osteopenia | 0.359 | | -1.48 | 2017.11.17 |
|  |  |  |  |  | |  |  |

**Supplementary Table 7D. Information about patients recruited for experiments**

| Aged  Osteoporosis | Gender | Age | Diagnosis | BMD  (g/cm^2^) | T value | Collection date |
| --- | --- | --- | --- | --- | --- | --- |
|  | male | 71 | Osteoporosis | 0.309 | -4.55 | 2017.10.28 |
|  | male | 69 | Osteoporosis | 0.39 | -3.2 | 2017.10.28 |
|  | male | 67 | Osteoporosis | 0.408 | -2.89 | 2017.11.12 |
|  | female | 69 | Osteoporosis | 0.266 | -3.52 | 2017.10.28 |
|  | female | 65 | Osteoporosis | 0.267 | -3.5 | 2017.10.28 |
|  | male | 68 | Osteoporosis | 0.391 | -3.17 | 2017.12.11 |
|  | female | 68 | Osteoporosis | 0.24 | -3.95 | 2017.11.13 |
|  | female | 72 | Osteoporosis | 0.317 | -2.66 | 2017.10.22 |
|  | male | 67 | Osteoporosis | 0.427 | -2.57 | 2017.10.21 |
|  | female | 67 | Osteoporosis | 0.249 | -3.79 | 2017.11.20 |
|  | female | 65 | Osteoporosis | 0.292 | -3.09 | 2017.12.1 |
|  | female | 65 | Osteoporosis | 0.273 | -3.4 | 2017.10.22 |
|  | female | 65 | Osteoporosis | 0.24 | -3.95 | 2017.10.28 |
|  | male | 63 | Osteoporosis | 0.353 | -3.81 | 2017.11.13 |
|  | male | 64 | Osteoporosis | 0.381 | -3.35 | 2017.10.22 |
|  | male | 63 | Osteoporosis | 0.385 | -3.28 | 2017.10.21 |
|  | male | 59 | Osteoporosis | 0.392 | -3.17 | 2017.11.20 |
|  | female | 62 | Osteoporosis | 0.26 | -3.61 | 2017.11.13 |
|  |  |  |  |  |  |  |
