# Supplementary material for: Involvement of serum‐derived exosomes of elderly patients with bone loss in failure of bone remodeling via alteration of exosomal bone‐related proteins
Source: Aging Cell. 2018 Mar 30;17(3):e12758. doi: 10.1111/acel.12758 (PMC5946082; doi:10.1111/acel.12758)
Supplement: Supplementary file 9 [file ACEL-17-e12758-s009.docx]

**Reagents**

Reagents and kits were purchased from commercial sources. The Total Exosome Isolation Reagent (from serum, 4478360), BCA protein assay kit (23227), TMT Mass Tagging Kits (90068) and fetal bovine serum (FBS, 10099-141) were purchased from Thermo Fisher Scientific (Waltham, MA, USA), and protease inhibitor cocktail (04693132001) was from Roche (Basel, Switzerland). Sequencing-grade endoproteinase Trypsin/Lys-C (V5073) was from Promega (Madison, WI, USA). Antibodies against CD63 (sc-15363) and ALIX (ab186429) were purchased from Santa Cruz Biotechnology (Dallas, TX, USA) and Abcam (Cambridge, UK), respectively. Iodoacetamide (IAA, RPN6302), dithiothreitol (DTT, 17-1318-01), urea (17-1319-01) and cell culture medium were from GE Healthcare (Little Chalfont, UK). mRANKL (462-TEC-010), hRANKL (6449-TEC-010), hM-CSF (216-MC-005), and hTGF-β1 (240-B-002) were purchased from R&D (Minneapolis, MN, USA). Ascorbic acid-2-phosphate (LAA, A4544), β-glycerol phosphate (β-GP, G9422), dexamethasone (D4902-25MG), tartrate-resistant acid phosphatase (TRAP) staining kits (387A), Alkaline phosphatase (ALP) assay kit (AP0100) and the Alizarin Red (A5533) were purchased from Sigma (St. Louis, MO, USA). An Xbridge BEH300 C18 column (4.6 × 250 mm, 5 μm) was obtained from Waters (Milford, MA, USA). A fused silica capillary column (75 μm ID, 150 mm length) was purchased from Upchurch (Oak Harbor, WA, USA), and C18 resin (300 A, 5 μm) was from Varian (Palo Alto, CA, USA).

**Tandem Mass Tag labeling**

Labeling was performed using the Tandem Mass Tagging (TMT) Kit following the manufacturer’s protocol with slight modifications. Equal amounts of protein (100 µg) from four groups were reduced with DTT and alkylated with IAA. Protein digestion was completed by incubation with Trypsin/Lys-C at a mass ratio of 1:25 (enzyme:protein) for 12 h at 37°C, following the manufacturer’s protocol. The digestion was terminated by heating at 60°C for 30 min. Digested proteins were then desalted, dried and finally dissolved in 200 mM triethylammonium bicarbonate buffer (pH 8.5). Different TM Tags were used to label the different groups: TMT-131 was used for the Osteoporosis patients group; TMT-130 for the Osteopenia group; TMT-127 for the Aged normal group; and TMT-126 for the Young normal group. After labeling, samples were pooled, desalted and dissolved in 0.1% trifluoroacetic acid (TFA).

**High performance liquid chromatography (HPLC) separation**

The TMT labeled peptides (100 μL in 0.1% TFA) were fractionated by HPLC (UltiMate 3000 UHPLC, Thermo Scientific) equipped with an Xbridge BEH300 C18 column maintained at 45°C. The peptides were eluted by a gradient acetonitrile elution buffer (pH 10) at a flow-rate of 1.0 mL/min. Each 1.5 mL elution was collected as one fraction. A total of 47 fractions were collected, dried and combined into 20 samples according to the peptide abundance. The samples were dissolved in 20 μL of 0.1% TFA for subsequent liquid chromatography (LC)-MS/MS analysis.

**Liquid chromatography (LC)-MS/MS analysis**

The LC-MS/MS analysis was performed as previously described[[1](#_ENREF_1), [2](#_ENREF_2)]. Briefly, peptides were first passed through a homemade fused silica capillary column (75 μm ID, 150 mm length; Upchurch) packed with C-18 resin (300 A, 5 μm; Varian, Lexington, MA) column on an EASY-nLC 1000 system using gradient TFA elution buffer (pH 1–2) at a flow-rate of 0.3 μL/min. The peptide eluate was ironed with a directly interfaced Thermo Orbitrap Fusion mass spectrometer (Thermo Scientific) in positive-ion mode. Xcalibur 3.0 software was used for mass data acquisition in a data-dependent mode.

**Data analysis**

Proteins were identified using Proteome Discoverer 2.1 software (Thermo Scientific) with the SEQUEST search engine. In detail, the raw MS data files were searched against the UniProt/SwissProt human proteome database (released on September 7, 2016). The following parameters were set as the search criteria: precursor mass tolerance, 20 ppm; fragment mass tolerance, 0.02 Da; total intensity threshold, 20,000; minimum number of peaks, 200; and a maximum of two missed cleavages was allowed. Carbamidomethylation (on C) and TMT 6-plex (on K and the peptide N-terminus) were set as static modifications, and oxidation (on M) was specified as a dynamic modification. Protein identification was considered valid if at least one peptide was statistically significant (*P* < 0.05 with a false discovery rate of 5%). The high-abundance serum proteins (albumin, IgG, antitrypsin, IgA, transferrin, haptoglobin, fibrinogen, alpha2-macroglobulin, alpha1-acid glycoprotein, IgM, apolipoprotein AI, apolipoprotein AII, complement C3, and transthyretin) were removed from the protein list. Depletion of high-abundance proteins from the human proteomic sample protein quantification was performed using the TMT 6-plex method. Reporter monoisotopic m/z was tuned according to the raw spectral data. Proteins were quantified using unique peptides.

**Protein** **identification using MS/MS data**

Representative MS/MS spectral identification was performed as previously described[[3](#_ENREF_3)]. Briefly, MS/MS spectral data of identified peptides and the intensity of TMT precursor ions were used for protein quantification. The masses of the resulting peptides were measured to obtain a TOF spectrum. Peaks from the TOF spectrum were selected for sequencing by fragmentation (MS/MS).

**Nanoparticle Tracking Analysis**

Exosomes purified from 100 uL serume with Total Exosome Isolation Reagent were resuspended in equal volume PBS and further diluted 25,000 fold for Nanoparticle tracking analysis (NTA). Approximately 300 μl of diluted exosomes were injected into the sample chamber of a Nanosight LM10 (Nanosight, Amesbury, UK). Nanoparticles were illuminated by a 635 nm laser and their movement under Brownian motion was record for 60 seconds. There videos were collected and analyzed with NTA 3.2 software (Nanosight, Amesbury, Wiltshire, UK). Data are presented as the average and standard deviation of the three video recordings.

**Human osteoblast differentiation**

The human fetal osteoblastic 1.19 cell line (hFOB 1.19) was obtained from Cell Bank of the Chinese Academy of Sciences (Shanghai, China). The hFOB 1.19 cell line was maintained in Dulbecco’s modified Eagle’s medium/Ham’s F-12 medium without phenol red (Invitrogen, Waltham, MA, USA), supplemented with 10% fetal bovine serum (Invitrogen, Waltham, MA, USA), 0.3 mg/ml G418/geneticin (Amresco, Radnor, PA, USA). For regular cell proliferation, cell was cultured at 33.5°C. For in vitro osteoblastic differentiation, hFOB 1.19 cells was cultured at 39.5°C, with addition of differentiation reagents: 100 μg/mL ascorbic acid, 10 mM β-glycerol phosphate, and 100 nM dexamethasone (all from Sigma).

**Human osteoclast differentiation from peripheral blood mononuclear cells (PBMCs)**

Human peripheral blood was provided by a male volunteer (40 years old) and a female volunteer (30 years old). PBMC was isolated from 50 mL whole blood with Ficoll-Paque PLUS (GE Healthcare, Chicago, IL, USA) according to the manufacture’s brochure. For osteoclast differentiation, PBMC was counted and place in 48-well plate at 6×105 cells per well in 0.5 ml of medium (α-MEM, containing 10% FCS). Medium was supplemented with 25 ng/ml human M-CSF, 50 ng/ml human RANKL, 5 ng/ml human TGF-β1, and 1 μM dexamethasone. The cells were re-fed twice weekly by demi-depletion (half of the medium withdrawn and replaced with the fresh medium).

**Mouse cell culture for osteoclast and osteoblast differentiation**

The RAW 264.7 cell line was obtained from the Chinese Academy of Medical Sciences (Beijing, China). Osteoclast formation was performed as previously described by Vincent *et al.*[[4](#_ENREF_4)], with minor modifications. Briefly, to generate mature multinucleated osteoclasts, RAW 264.7 cells (1.5×10^5^ cells/cm^2^) were cultured in α-minimum essential medium (α-MEM) with 10% FBS and an additional 10 ng/mL RANKL in 6-well plates and incubated at 37°C in 5% CO_2_. For osteoblast differentiation, MC3T3-E1 cells were induced with differentiation medium (DM), comprising the above differentiation medium (GM) supplemented with 50 μg/mL LAA and 10 µM β-GP. DM was replaced every 2–3 days.

**TRAP, Bone resorption, Alkaline phosphatase (ALP) assay and Alizarin Red staining**

Mature osteoclasts were defined as TRAP-positive cells containing three or more nuclei. TRAP staining was performed using the TRAP staining kit according to the manufacturer’s instructions. Briefly, cells were washed three times with PBS and fixed with fixative solution (65% acetone and 3.7 % formaldehyde in citrate solution). Fixative solution was completely removed and cells were washed three times with ddH2O. Cells were stained with TRAP solution for 1 h at 37°C in the dark. For bone resorption assays, RAW264.7 or human PBMC cells were seeded on Osteo Assay Surface (Corning, New York, USA) and stimulated with differentiation reagents for 5 days. After remove the cells, the Osteo Assay Surface was stained with 1% toluidine blue. After stimulating with differentiation reagents for 21 days, hFOB1.19 cells were solved in lysis buffer (50 mM Tris-HCl, pH 7.4, 0.1% Triton X-100). ALP activity was measured using ALP detection kit (AP0100, Sigma) according to the manufacturer's instructions. The absorption value was detected with Varioskan™ Flash microplate reader (Waltham, MA, USA) at 405 nm wavelength. Protein concentration was measured with BCA protein assay kit. ALP activity was shown as 10-3Unit per mg protein. Mineralization in osteogenic cultures was determined by Alizarin Red staining. Cells were washed with PBS, fixed with 3.7% formaldehyde for 10 min, washed three times with PBS, and then stained with 40 mM Alizarin Red (pH 4.1) for 15 min at room temperature. The cultures were washed three times with ddH2O and photographed under a light microscope. For matrix mineralization quantification, calcium-bound Alizarin Red was solubilized with 10% acetic acid for 30 min. The supernatant was then collected and ammonium hydroxide was added to pH 4.1. The degree of mineralization was indicated by supernatant absorbance at 405 nm measured using a spectrophotometer.

**Western blotting analysis**

Exosome markers CD63 and ALIX were probed using specific antibodies. For Western blot analysis, the reagent-isolated exosomes dissolved in PBS were first lysed by RIPA buffer. The lysed exosomes (20 µg total protein) were then separated by SDS-PAGE. Gels were run according to standard methods and proteins were electrophoretically transferred to nitrocellulose membranes. Membranes were blocked with 5% non-fat dried milk in TBS-T (TBS plus 0.05% Tween-20) for 30 min. The membranes were then incubated with primary antibodies (diluted 1:200 for CD63 and ALIX) overnight at 4°C. Membranes were washed and incubated with horseradish peroxidase-conjugated secondary antibodies and ECL reagents. Coomassie Brilliant Blue staining was used as a control to assess standardized loading.

**Enzyme-linked immunosorbent assay (ELISA)**

For each target protein detection, 100 uL serum derived exosomes were resuspended in 100 uL ELISA lysis buffer (100 mM Tris-HCl, pH7.4, 150 mM NaCl, 1 mM EGTA, 1 mM EDTA, 1% Triton X-100, 0.5% Sodium deoxycholate, added with proteases inhibitor cocktail immediately before use). Experiment procedure was performed following instruction with no modification. Human ITGB1 ELISA kit and Human ITGB3 ELISA kit were both purchased from CUSABIO (Wuhan, China).

[1] Bie Y, Zhang Z. RAB8A a new biomarker for endometrial cancer? World journal of surgical oncology. 2014;12:371.

[2] Kim J, Tan Z, Lubman DM. Exosome enrichment of human serum using multiple cycles of centrifugation. Electrophoresis. 2015;36:2017-26.

[3] Cottrell JS. Protein identification using MS/MS data. Journal of proteomics. 2011;74:1842-51.

[4] Vincent C, Kogawa M, Findlay DM, Atkins GJ. The generation of osteoclasts from RAW 264.7 precursors in defined, serum-free conditions. Journal of bone and mineral metabolism. 2009;27:114-9.
